# Identification key to the *Anopheles* mosquitoes of South America (Diptera: Culicidae). III. Male genitalia

**DOI:** 10.1186/s13071-020-04300-1

**Published:** 2020-10-31

**Authors:** Maria Anice Mureb Sallum, Ranulfo González Obando, Nancy Carrejo, Richard C. Wilkerson

**Affiliations:** 1 Departamento de Epidemiologia, Faculdade de Saúde Pública, Universidade de São Paulo, Avenida Doutor Arnaldo 715, São Paulo, São Paulo CEP01246-904 Brazil; 2 Departamento de Biología, Universidad del Valle, A.A 25360 Cali, Colombia; 3 Department of Entomology, Smithsonian Institution, National Museum of Natural History (NMNH), Washington, DC 20560 USA; 4 Walter Reed Biosystematics Unit, Smithsonian Institution Museum Support Center, 4210 Silver Hill Rd., Suitland, MD 20746 USA; 5 Walter Reed Army Institute of Research, 503 Robert Grant Avenue, Silver Spring, MD 20910 USA

**Keywords:** *Anopheles*, Illustrated key, Male genitalia, Morphology, South America

## Abstract

**Background:**

Accurate identification of the species of *Anopheles* Meigen, 1818 requires careful examination of all life stages. However, morphological characters, especially those of the females and fourth-instar larvae, show some degree of polymorphism and overlap among members of species complexes, and sometimes even within progenies. Characters of the male genitalia are structural and allow accurate identification of the majority of species, excluding only those in the Albitarsis Complex. In this key, based on the morphology of the male genitalia, traditionally used important characters are exploited together with additional characters that allow robust identification of male *Anopheles* mosquitoes in South America.

**Methods:**

Morphological characters of the male genitalia of South American species of the genus *Anopheles* were examined and employed to construct a comprehensive, illustrated identification key. For those species for which specimens were not available, illustrations were based on published illustrations. Photographs of key characters of the genitalia were obtained using a digital Canon Eos T3i attached to a light Diaplan Leitz microscope. The program Helicon Focus was used to build single in-focus images by stacking multiple images of the same structure.

**Results:**

An illustrated key to South American species of *Anopheles* based on the morphology of the male genitalia is presented, together with a glossary of morphological terms. The male genitalia of type-specimens of previously poorly documented species were also examined and included in the key, e.g. *Anopheles* (*Anopheles*) *tibiamaculatus* (Neiva, 1906) which has a unique quadrangular-shaped aedeagus with an apical opening.

**Conclusions:**

Male genitalia of South American species of *Anopheles* possess robust characters that can be exploited for accurate species identification. Distortion that can occur during the dissection and mounting process can obstruct accurate identification; this is most evident with inadvertent damage or destruction of unique features and interferes with correctly assigning shapes of the features of the ventral claspette. In some species, the shape, and anatomical details of the aedeagus also need to be examined for species identification. For members of the Myzorhynchella Series, both ventral and dorsal claspettes possess multiple characteristics that are herein used as reliable characters for species identification.
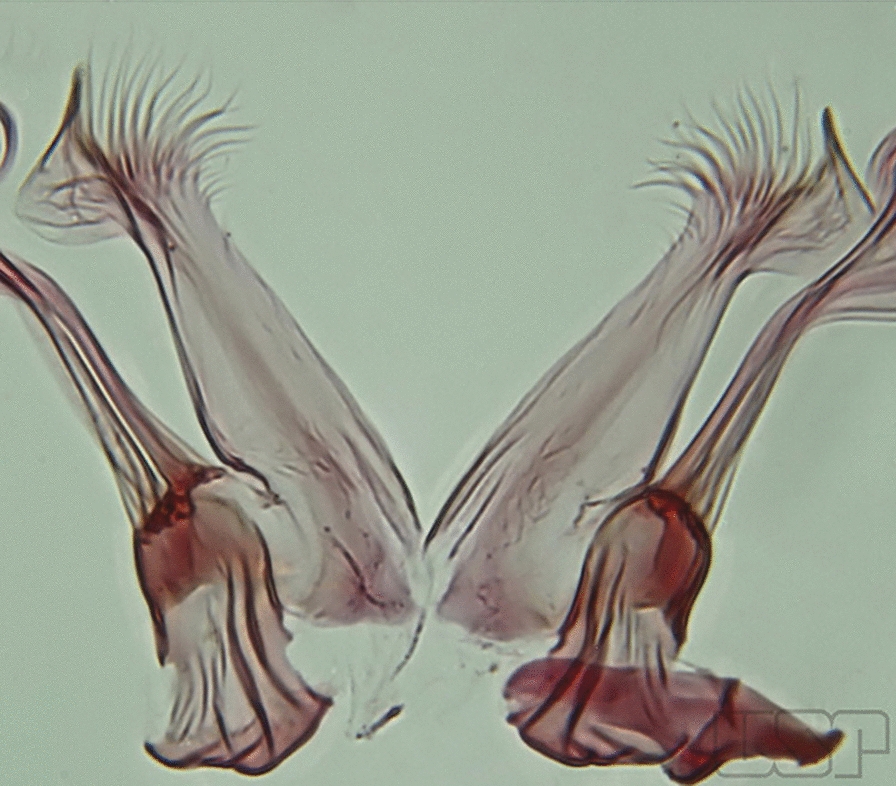

## Background

General introductory comments, distributions and species authors and publication dates are given in Part I [1] of this series of four articles. Keys to the fourth-instar larvae and adult females are provided in Parts II [2] and IV [3], respectively. A list of species treated here is included in Part I [1].

## Methods

The primary types (holotypes and paratypes) and other field-collected specimens deposited in the Coleção Entomológica de Referência, Faculdade de Saúde Pública, Universidade de São Paulo, São Paulo, Brazil (FSP-USP), Museo de Entomología, Universidad del Valle, Santiago de Cali, Colombia (MUSENUV) and the US National Mosquito Collection, Smithsonian Institution, Washington, DC, USA (USNMC) were examined to discover characters to be used in the male genitalia key. For species that we could not access, illustrations were based on published illustrations. Photomicrographs of relevant characters for the male genitalia were taken using a digital Canon Eos T3i (Canon, USA), attached to a Diaplan Leitz microscope, using the program Helicon Focus software (https://www.heliconsoft.com/heliconsoft-products/helicon-focus/), which was used to build single in-focus images by stacking multiple images of the same structure. Photomicrographs were further processed in Adobe Photoshop (https://www.photoshop.com/en) to embed names and labels. The institutional sources of specimens are recorded on each photograph. The nomenclature adopted is that of Harbach & Knight [4, 5]. The water marks embedded in photomicrographs of the male genitalia show the institution where the vouchers are deposited, Universidade de Sao Paulo (USP) and Universidad del Valle.

The key includes most of the species registered in South America, except for 15 species that are poorly known. They are the following: *Anopheles* (*Ano*.) *annulipalpis* Lynch Arribálzaga; *An*. (*Ano*.) *bustamantei* Galvão; *An*. (*Ano*.) *evandroi* da Costa Lima; *An*. (*Ano*.) *pseudomaculipes* (Chagas); *An*. (*Ano*.) *pseudopunctipennis levicastilloi* Leví-Castillo; *An.* (*Ano*.) *pseudopunctipennis neghmei* Mann; *An.* (*Ano*.) *pseudopunctipennis noei* Mann; *An.* (*Ano*.) *pseudopunctipennis patersoni* Alvarado & Heredia; *An*. (*Ano*.) *pseudopunctipennis rivadeneirai* Leví-Castillo; *An*. (*Ano*.) *rachoui* Galvão; *An*. (*Ker*.) *auyantepuiensis* Harbach & Navarro; *An*. (*Ker*.) *boliviensis* (Theobald); *An*. (*Ker*.) *rollai* Cova García, Pulido F. & Escalante de Ugueto; *An*. (*Nys*.) *nigritarsis* (Chagas); and *An*. (*Nys*.) *sanctielii* Senevet & Abonnenc. For these species, it will be necessary to conduct field collections in the type-localities and further taxonomic investigations.

## Results and discussion

### Glossary of morphological terms

The terminology of the male genitalia used in this key follows that of Harbach & Knight [4, 5]. Also known as the male terminalia, Harbach & Knight [4] recommended instead to use “the genitalia” to avoid confusion with other terminal structures. The composite male genitalia are structures formed from elements of the posterior segments IX and X of the abdomen. These modified structures are involved in mating, copulation, and insemination. After emergence of the adult male, the posterior part of the abdomen beyond segment VII makes a 180° rotation. Thus, the ventral segmental surfaces become dorsal in relation to the rest of the abdomen, and *vice versa*. In Culicidae Meigen, 1818, the male genitalia are therefore inverted in relation to the female genitalia. This means that when coupling occurs, both individuals have the same upright orientation, instead of the male ending up vulnerable, and upside down in relation to the female, as is the case in the family Tabanidae Latreille, 1802 (horse flies), which do not have male genital rotation [6]. This phenomenon must be considered when describing the position of the various elements of the genitalia.

Tergum IX, which usually varies little, can exhibit useful morphological variation in certain species. Species of the Arribalzagia Series of the subgenus *Anopheles* Meigen, 1818 possess ninth tergal lobes (IX-Te lobes) of variable size and development, features which can be useful for species recognition. The internal margin of tergum IX is attached to the proctiger that is formed by tergum X, the cerci, the cercal sclerites and the paraprocts. Dorsally there are two sclerotized plates called the cercal sclerites. The two structures attached laterally on the most posterior part of the abdomen are called the gonocoxopodites. They are adapted to facilitate insemination by grasping the female during copulation. The gonocoxopodites are composed of a proximal gonocoxite and a distal gonostylus.

**Fig. 1:**
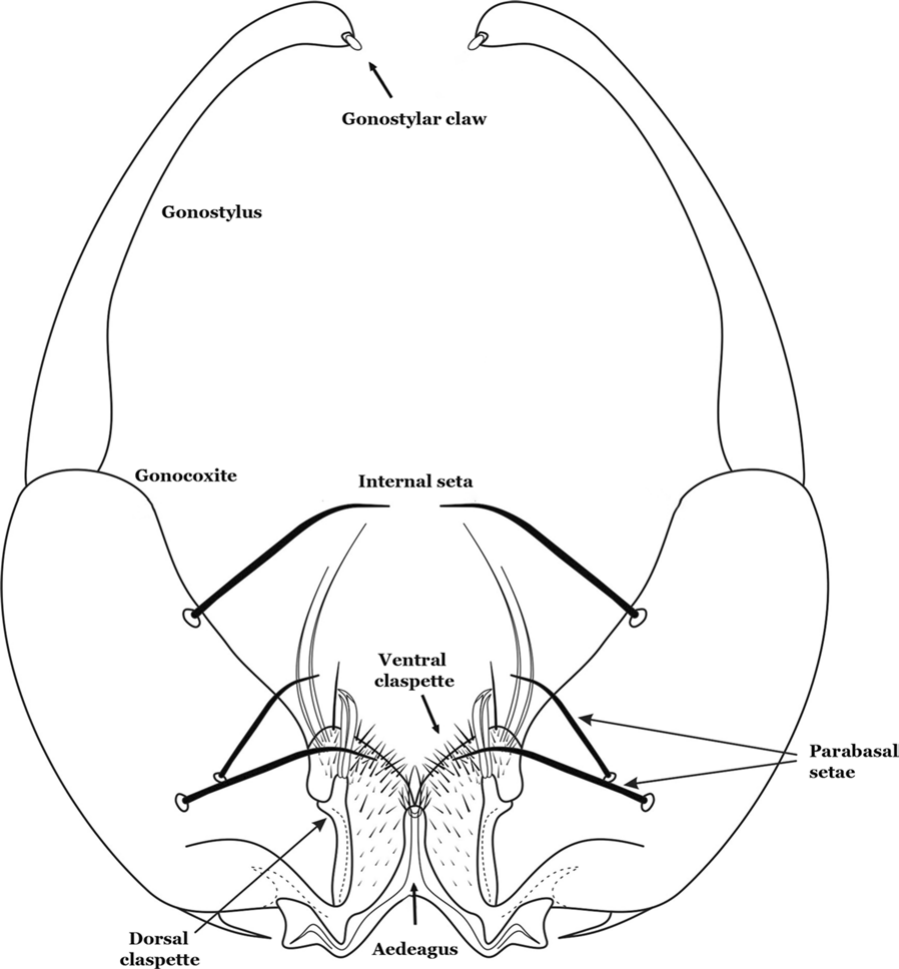
Schematic drawing of the male genitalia of *An*. *pseudopunctipennis* Theobald, 1901. The proctiger (anal lobe) was removed to permit an unobstructed view of the ventral structures (redrawn after Komp [7])

The gonocoxite (Fig. 1) is a large, relatively long, and somewhat conical structure, much wider than the gonostylus. The external ventral surface is convex, while the internal surface is slightly concave, especially basally at the attachment of the claspettes [7]. The gonocoxites bear a large number of scales and setae, the larger of which are sometimes called spines; for purposes of this key, the term seta will be used. One or two parabasal setae are inserted on the dorsobasal portion of the gonocoxite. In species of the subgenera *Nyssorhynchus* Blanchard, 1902 and *Kerteszia* Theobald, 1905, the setae are inserted on prominent parabasal lobes that are situated dorsobasally (in a prerotational sense). The parabasal lobes are absent in Neotropical species belonging to the subgenera *Anopheles*, *Lophopodomyia* Antunes, 1937 and *Stethomyia* Theobald, 1902. The parabasal setae are instead inserted directly on the surface or on relatively small projections on the surface of the gonocoxite. In species of the subgenus *Stethomyia*, the parabasal setae are absent.

**Fig. 2:**
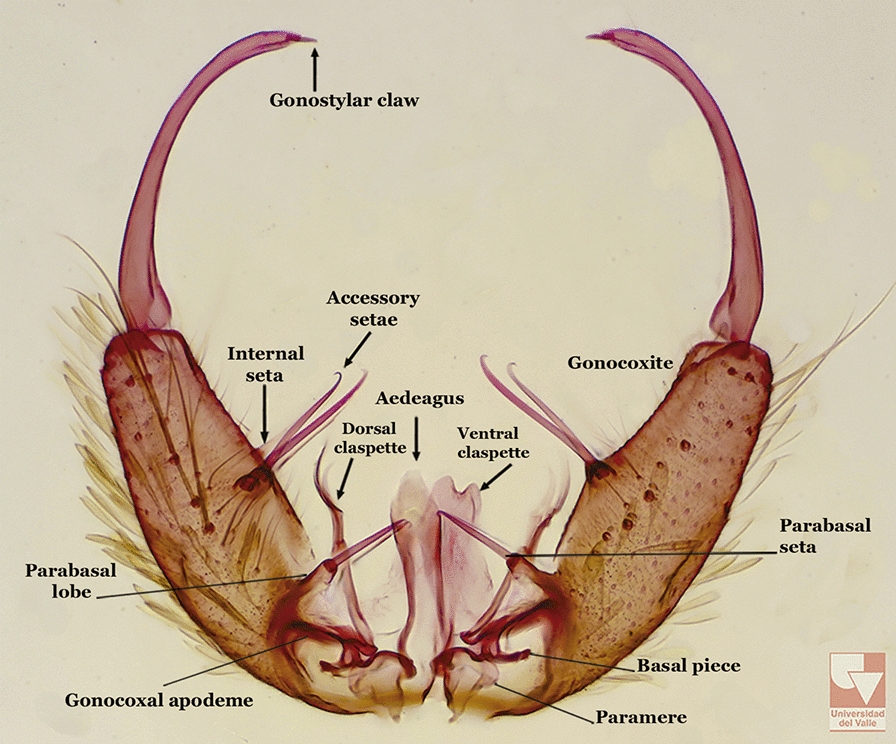
Dorsal view of the male genitalia of *An*. *albimanus* Wiedemann. Segment IX and the proctiger (anal lobe) were removed to facilitate the observation of structures that occupy the ventral position

**Fig. 3:**
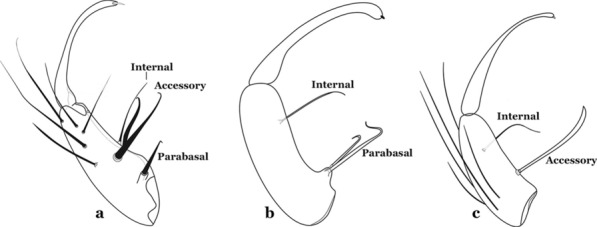
Gonocoxites showing the accessory setae, internal seta and parabasal setae of species of the subgenera *Nyssorhynchus* (**a**), *Anopheles* (**b**), *Stethomyia* (**c**). **b** and **c** redrawn after Komp [7]

Species of the subgenera *Nyssorhynchus* (Fig. 2) and *Kerteszia* have a single dorsally directed seta that is inserted on the parabasal lobe. The Neotropical species of the subgenera *Anopheles* and *Lophopodomyia* have two parabasal setae (Fig. 3b, c). The internal seta is inserted on the ventral surface of the gonocoxite, on the distal half or near mid-length. Species of the subgenera *Nyssorhynchus* and *Kerteszia* have a pair of accessory setae inserted on the dorsal surface of the gonocoxite (Figs. 2, 3a).

The gonostylus corresponds to the stylus of the gonocoxopodite. It is a well-sclerotized structure, moveable and articulated, on or near the apex of the gonocoxite. It is somewhat thickened and curved. At its apex is a small spiniform structure called the gonostylar claw [8] (Fig. 3).

**Fig. 4:**
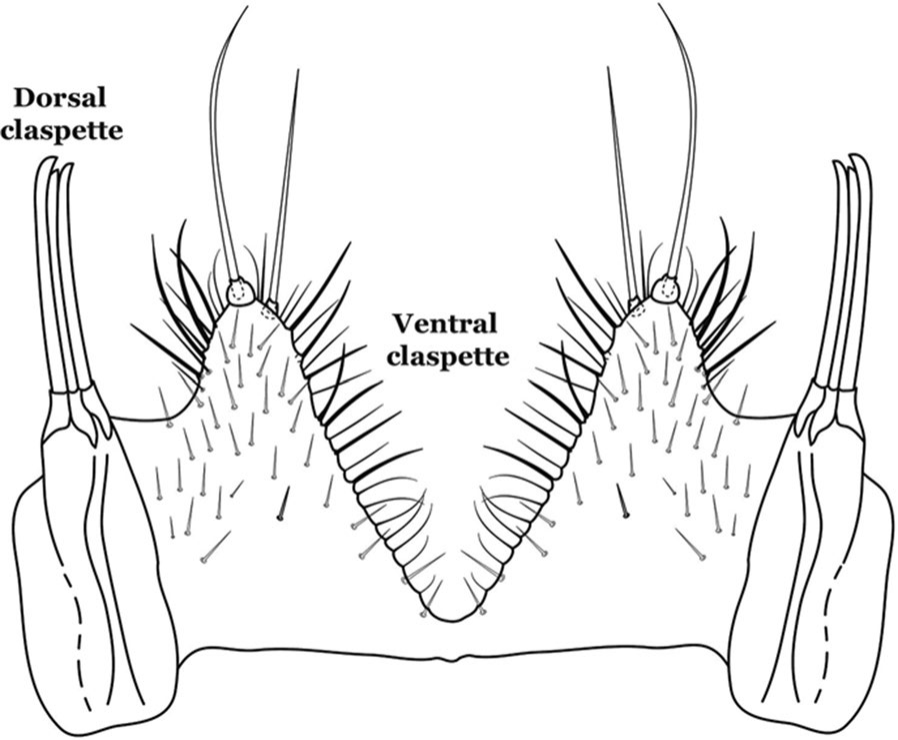
Ventral and dorsal lobe claspettes of the male of *An*. *neomaculipalpus* Curry, 1931(redrawn after Komp [7])

Attached to the internal surface of the gonocoxite is the claspette. This is a membranous structure, usually divided into ventral and dorsal lobes, both exhibiting great variability according to subgenus and species within the subgenera (Fig. 4). For purposes of this key, the ventral and dorsal lobes of the claspette [4] are referred to as the ventral claspette and dorsal claspette, respectively. These are terms also used by Faran [9].

In species of the subgenera *Anopheles*, *Lophopodomyia* and *Kerteszia*, the dorsal claspette is divided into ventral and dorsal lobes. These lobes exhibit interspecific variability in the form of the setae, as well as the size and distribution of spicules, which makes them useful in taxonomy. In species of the subgenera *Anopheles* (Fig. 4), *Lophopodomyia* and *Kerteszia*, the ventral lobe is divided apically, with the distance between them being more pronounced in species of the subgenera *Kerteszia*. In species of the subgenus *Nyssorhynchus*, the ventral claspette is not subdivided, instead the two ventral claspettes are fused and the composite structure occupies a median position between the gonocoxites. The structure is rich in morphological variation and is therefore useful for species identification. In some taxa, the ventral claspette is smooth (i.e. without spicules) while in others the spicules can be short or long and variously distributed on the claspette.

**Fig. 5:**
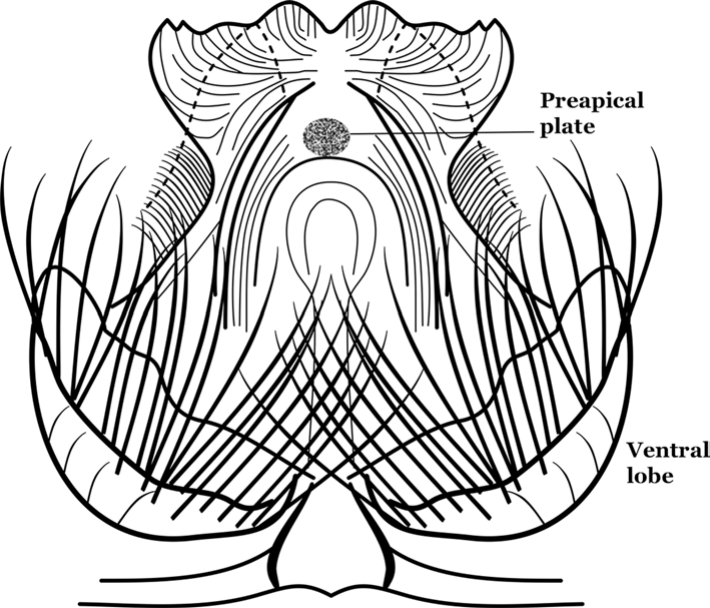
Ventral claspette of *An*. *strodei* Root, 1926 (redrawn after Faran & Linthicum [10])

**Fig. 6:**
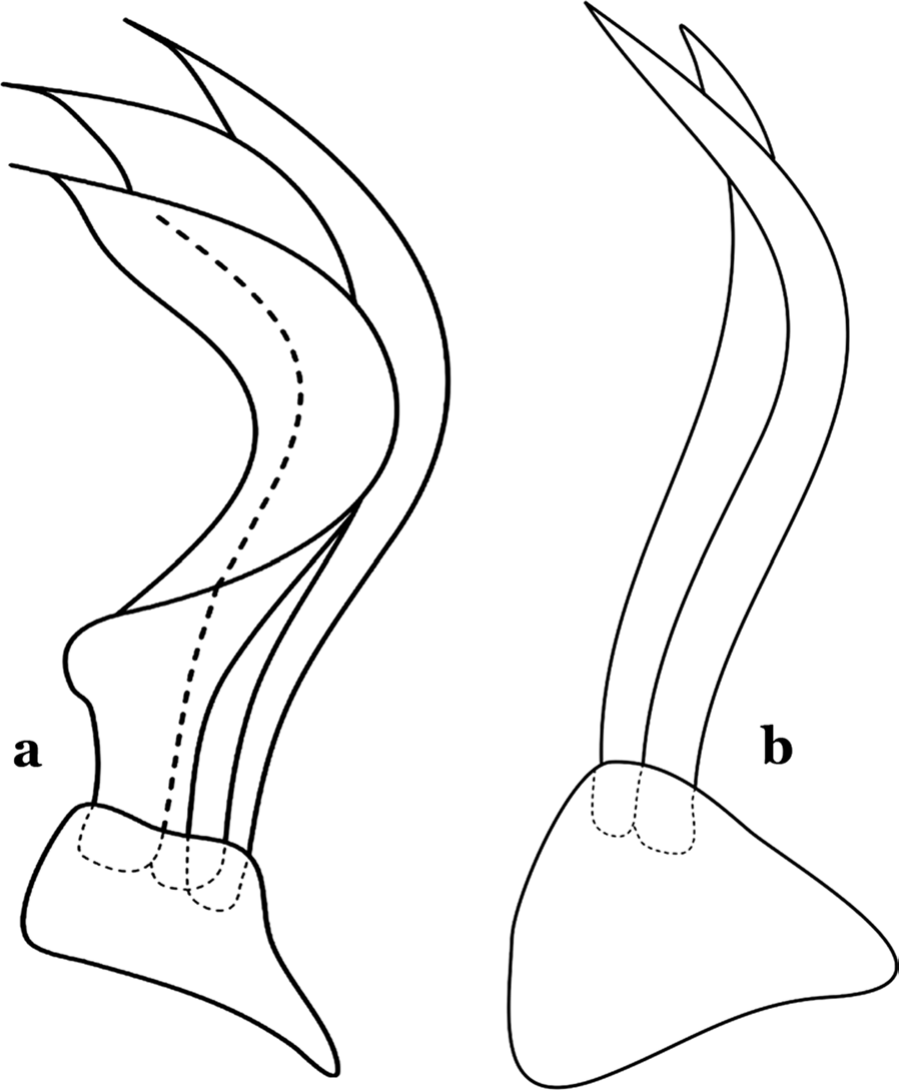
Basal portion of dorsal claspette of species of *Anopheles* (*Nyssorhynchus*). **a** *An*. *braziliensis* (Chagas, 1907). **b** *An*. *argyritarsis* Robineau-Desvoidy, 1827 (redrawn after Faran & Linthicum [10])

The apex of the ventral claspette can be rounded, truncated or angular, and, in some species can bear apicolateral expansions that resemble lobes. The presence of these apicolateral lobes in *An*. (*Nys*.) *triannulatus* (Neiva & Pinto, 1922) allows separation of this species from the otherwise morphologically similar *An*. (*Nys*.) *halophylus* Silva-do- Nascimento & Lourenço-de-Oliveira, 2002. Other structures that make up parts of the ventral claspette and are employed in taxonomy treatments include the preapical plate and basoventral lobes (Fig. 5). In species of the subgenera *Nyssorhynchus*, *Kerteszia*, *Anopheles* and *Lophopodomyia*, there is a short basal portion on the dorsal claspette upon which variable numbers of setae are attached dorsoventrally. These setae are variable in form, point of insertion, development, and quantity (Fig. 6). In species of the subgenus *Stethomyia*, the dorsal claspette is absent and the ventral claspette is columnar, with two subdivisions that support apical setae that are variously developed [7].

**Fig. 7:**
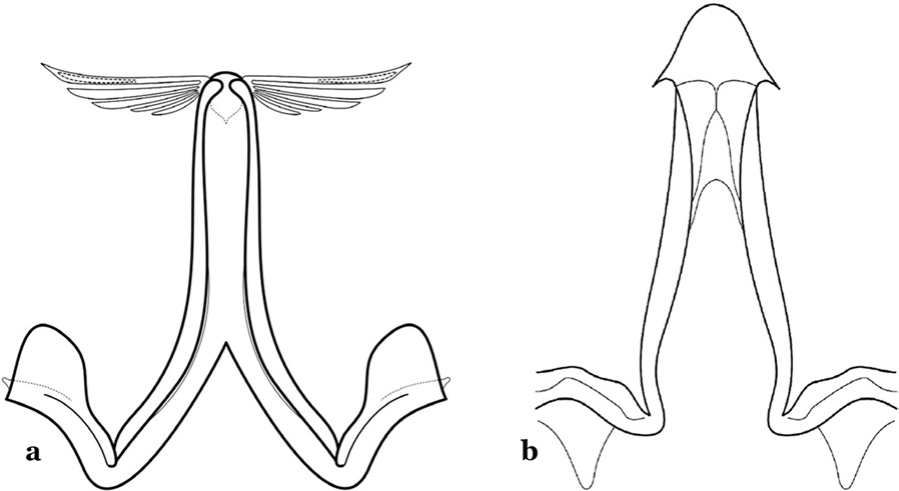
Aedeagus of the male genitalia. **a** Subgenus *Anopheles.* **b** Subgenus *Nyssorhynchus* (redrawn after Komp [7])

The aedeagus is part of the phallosome, which includes, in addition to the aedeagus, the parameres and the basal pieces. The aedeagus is articulated basally to the parameres, which are connected to the basal pieces by an acetabulum that is on the median lateral area. The basal pieces, responsible for movement of the aedeagus during copulation, are connected to the gonocoxal apodemes. The aedeagus is the central organ of the phallosome and serves as the intromittent organ [4]. In species of *Anopheles*, the aedeagus is a tubular structure, dorsally curved, with the walls unequally sclerotized and with a circular opening near or at the apex. The apical part of the aedeagus is variable in form and development. In species of the subgenus *Nyssorhynchus*, the apical part of the aedeagus is variable, and often used in species identification. The presence of leaflets subapically on the aedeagus, as well as the number of these structures, their form, development, and presence of marginal serrations, permit identification of many species of the genus (Fig. 7).

In species of the subgenus *Anopheles*, the apical leaflets, when present, can vary in number, position, form, and development. Some species possess a single pair of leaflets that can have smooth or serrate margins, be uniformly or unevenly sclerotized, and be short or long. In species of the subgenera *Kerteszia* and *Nyssorhynchus*, the leaflets may be present or absent. When present, they occur as a single subapical pair.

The morphological key using the external characters of the male genitalia can aid in identifying species of the genus *Anopheles* of the South America. Unnamed species of the known complexes can be identified as morphologically similar valid species. In the key, species complex is labelled as (*s*.*l*.). For these groups, further investigations will be necessary to define characters of the male genitalia for accurate identification. The key was modified from [10, 11, 13, 14], with additional characters provided herein.

### Key for the identification of species of the genus *Anopheles* of South America based on characters of the male genitalia

**Fig. 8:**
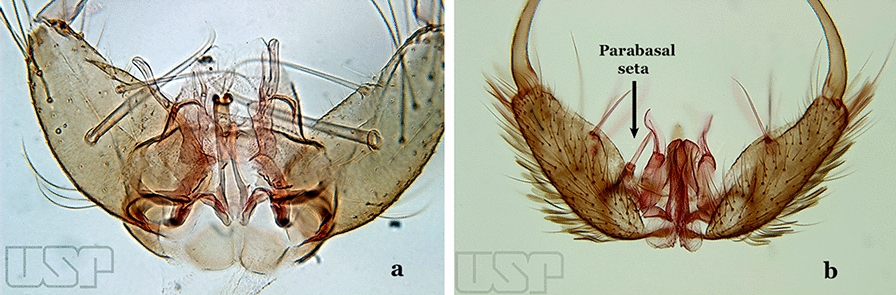
**a***An*. *thomasi* Shannon, 1933. **b** *An*. *antunesi* Galvao & Franco do Amaral, 1940
- Gonocoxite with 1 or 2 parabasal setae (Fig. 8b)…..6
- Ventral claspette with 2 spiniform setae (Fig. 9a)…..3

- Gonocoxite without parabasal setae (Fig. 8a)…..2

**Fig. 9:**
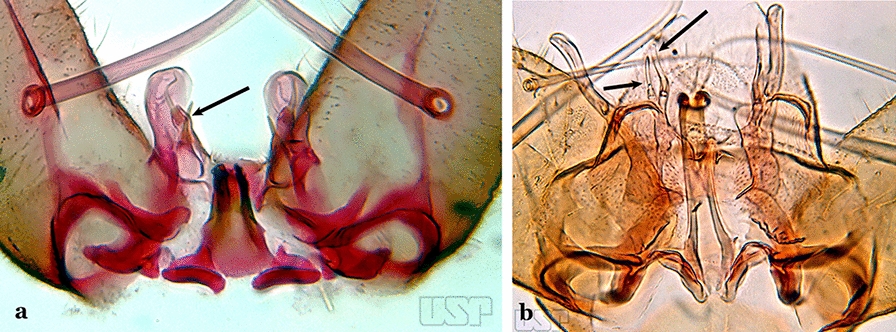
**a***An*. *kompi* Edwards, 1930. **b** *An*. *thomasi*
- Ventral claspette with 1 spatulate and 1 spiniform seta (Fig. 9b)…..4
- Accessory seta inserted on proximal third of gonocoxite (Fig. 10)….. *An*. *kompi*

**Fig. 10:**
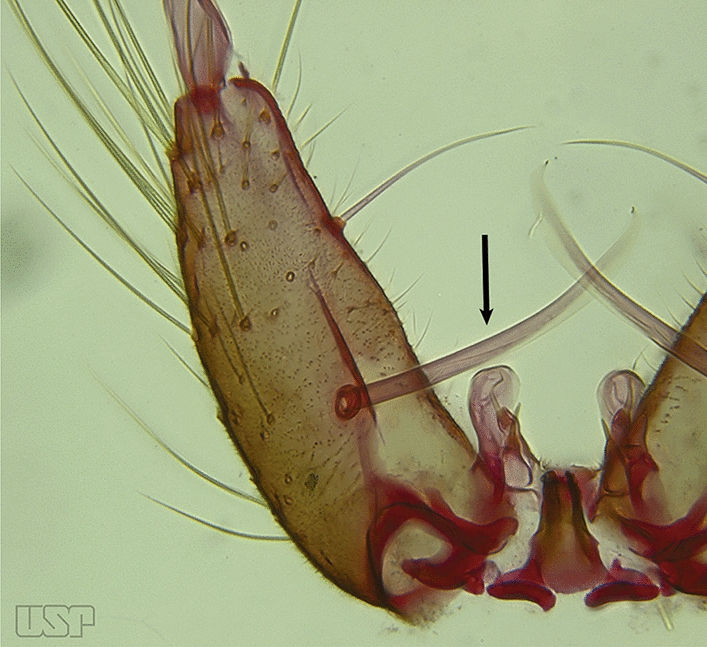
*An*. *kompi*
- Accessory seta inserted near middle of gonocoxite….. *An*. *canorii*
- Dorsal claspette with longest subdivision lacking a subapical projection in form of a beak (Fig. 11a)…..*An*. *thomasi*

**Fig. 11:**
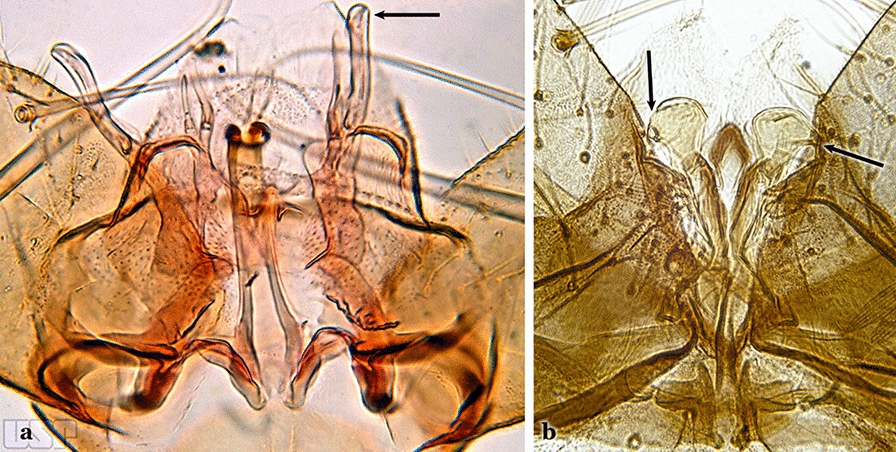
**a***An*. *thomasi*. **b***An*. *nimbus* (Theobald, 1902)
- Dorsal claspette with longest subdivision having a subapical projection in form of a long or short beak (Fig. 11b)…..5
- Dorsal claspette with longest subdivision having a subapical projection in form of a long beak (Fig. 12a)….. *An*. *acanthotorynus*

**Fig. 12:**
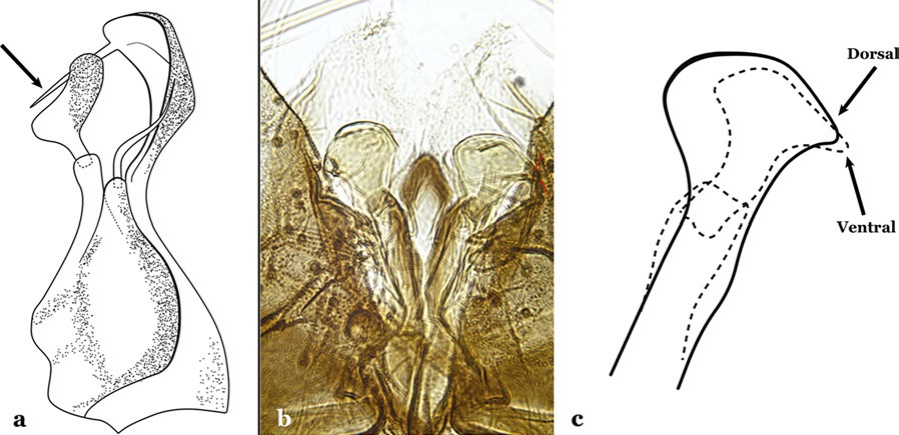
**a***An*. *acanthotorynus* Komp, 1937 (redrawn after Komp [12]). **b, c** *An*. *nimbus* (**c** drawn from **b**)
- Dorsal claspette with longest subdivision having a subapical projection in form of a short beak (Fig. 12b)…..*An*. *nimbus*
- Gonocoxite with 2 accessory setae and 1 internal seta (Fig. 13a)…..7

**Fig. 13:**
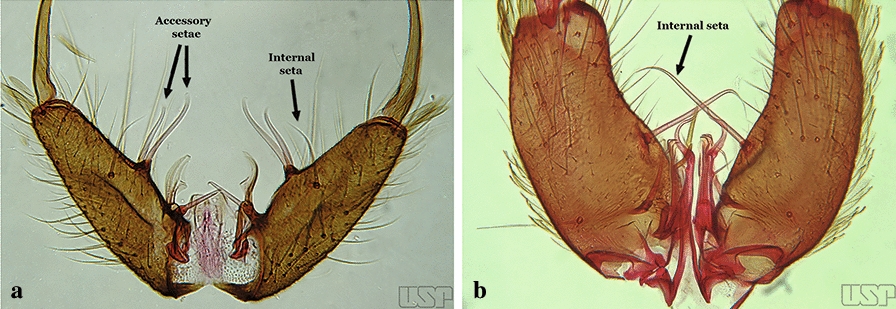
**a***An*. *braziliensis*. **b***An*. *costai* da Fonseca & da Silva Ramos, 1940
- Gonocoxite without accessory setae, internal seta present or absent (Fig. 13b)…..53
- Gonocoxite with parabasal seta inserted on a basodorsal lobe (Fig. 14a)…..8

**Fig. 14:**
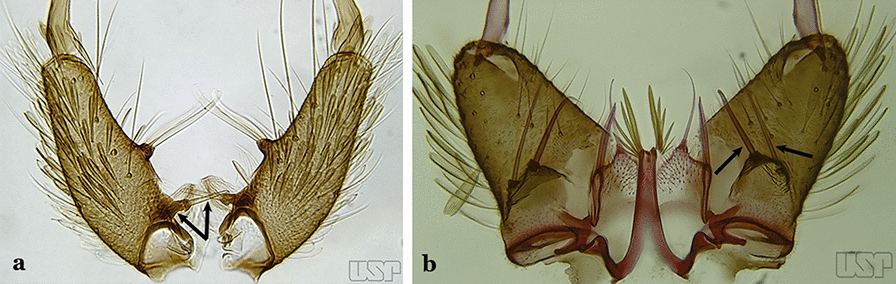
**a***An*. *darlingi* Root, 1926. **b** *An*. *peryassui* Dyar & Knab, 1908
- Gonocoxite with parabasal seta inserted directly on the surface of its proximal third (Fig. 14b)…..48
- Parabasal seta long and curved, apex truncate; internal seta inserted between accessory and parabasal setae (Fig. 15a); sternum IX with a median longitudinal apodeme (Fig. 15b) (*Kerteszia*)…..9

**Fig. 15:**
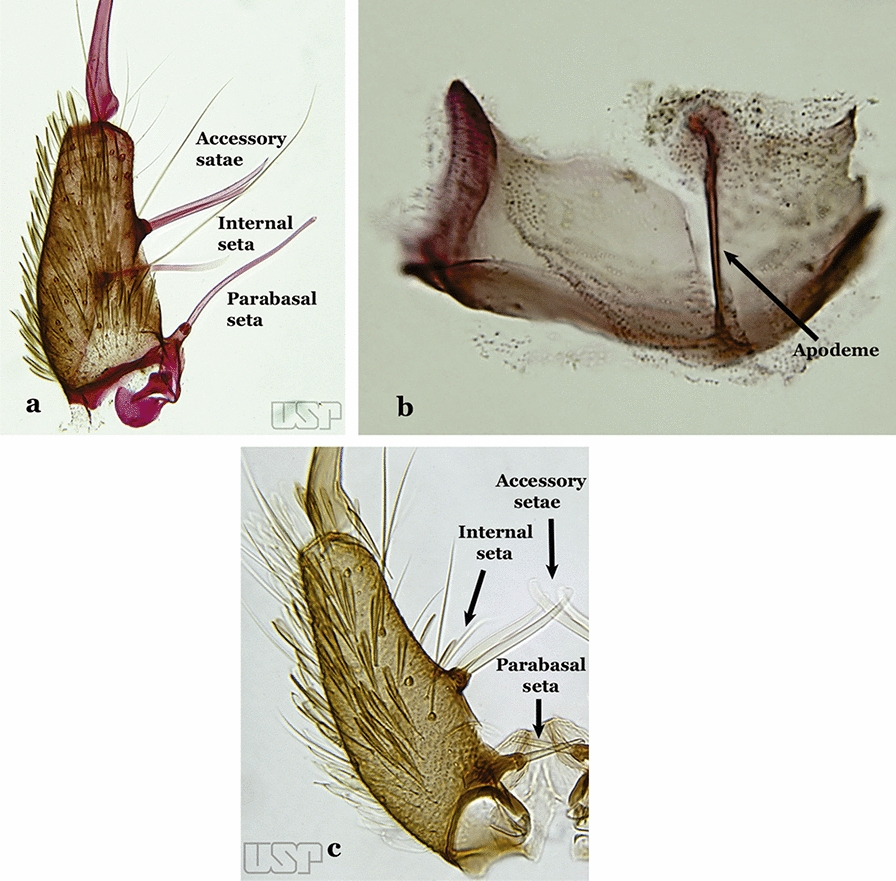
**a**, **b***An*. *homunculus* Komp, 1937. **c** *An*. *darlingi* Root, 1926
- Parabasal seta short and strong, apex ending in a small hook or tapering to apex; accessory setae inserted between internal and parabasal setae (Fig. 15c); sternum IX without a median longitudinal apodeme (*Nyssorhynchus*)…..17
- Aedeagus without leaflets (Fig. 16a)…..10

**Fig. 16:**
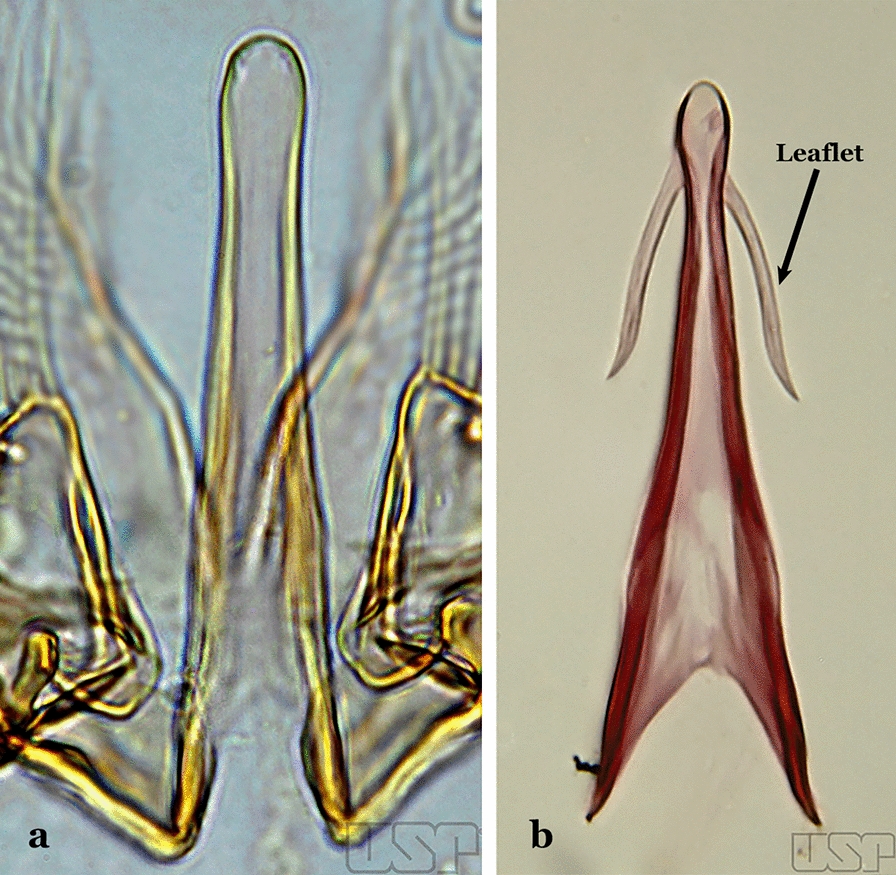
Aedeagus of *An*. *neivai* (Lane & Coutinho, 1940) (**a**) and *An*. *cruzii* Dyar & Knab, 1908 (**b**)
- Aedeagus with a pair of subapical leaflets (Fig. 16b)…..14
- Ventral claspette smooth except for 4–11 strong spicules, and/or less developed spicules along median margin (Fig. 17a)…..*An*. *bambusicolus*

**Fig. 17:**
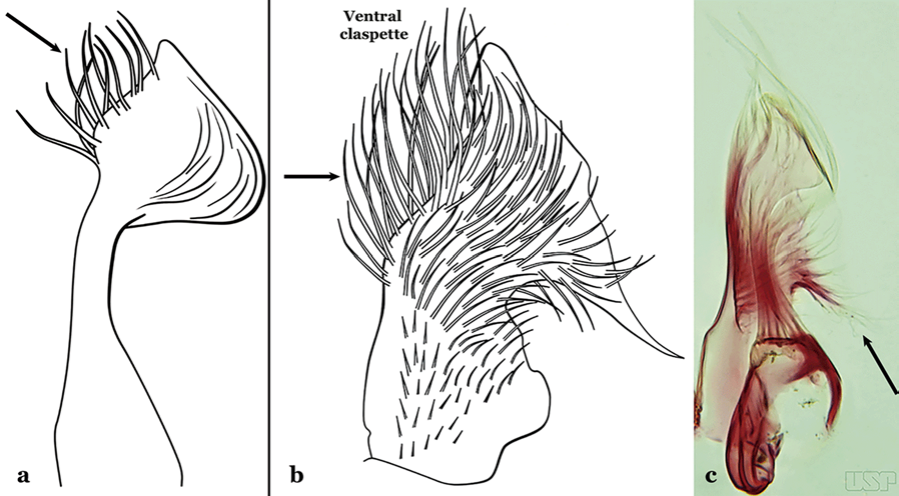
**a** Ventral claspette of *An*. *bambusicolus* Komp, 1937. **b, c** *An*. *homunculus* (**b** redrawn after Zavortink [13])
- Ventral claspette moderately or densely spiculose, spicules distributed over its entire surface (Fig. 17b)…..11
- Tergum VIII without broad median scales (Fig. 18)…..12

**Fig. 18:**
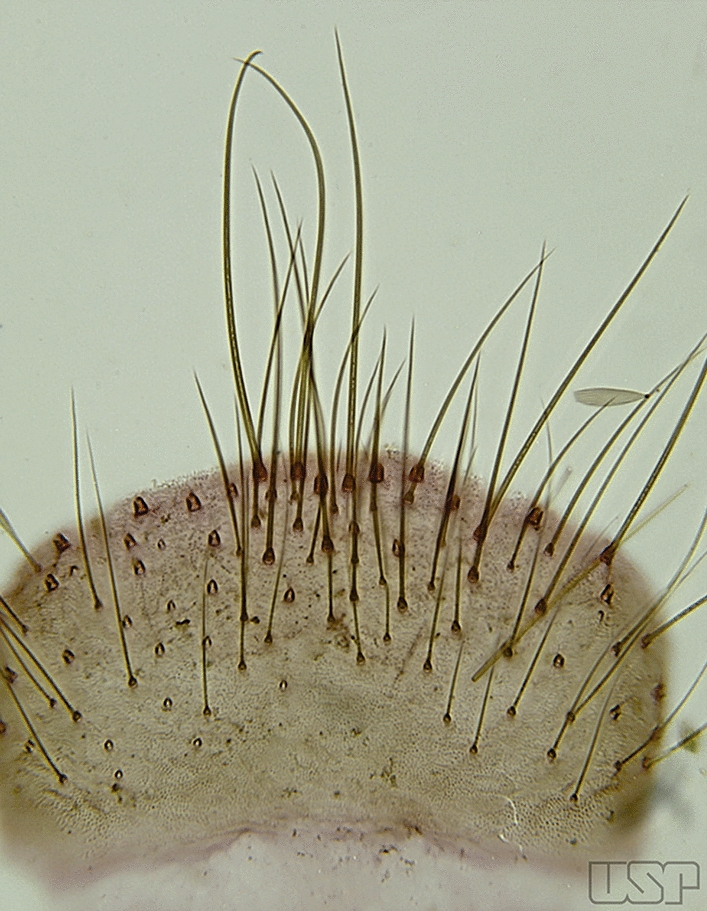
*An*. *homunculus*
- Tergum VIII with numerous broad median scales…..13
- Ventral claspette with a straight lateral expansion, not curved ventroposteriorly; gonocoxite with internal seta flattened, wider near apex (Fig. 19a)….. *An*. *neivai* (*s.l.*)

**Fig. 19:**
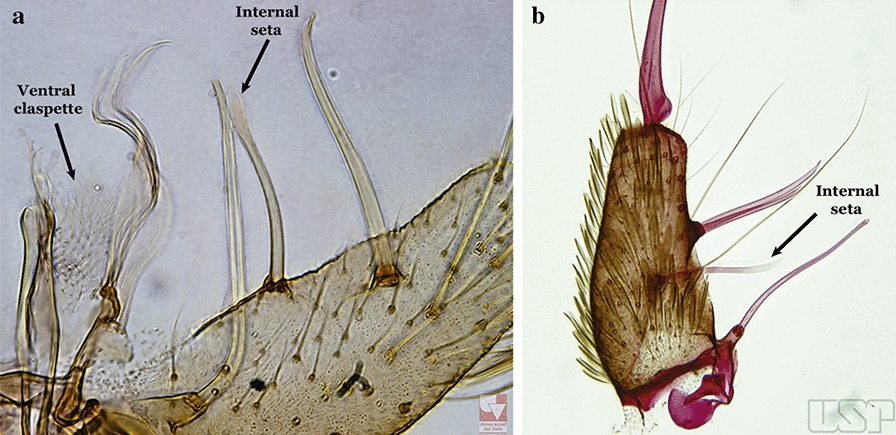
**a***An*. *neivai*. **b***An*. *homunculus*
- Ventral claspette with a large lateral expansion, curved ventroposteriorly, forming a sharp point directed anteriorly (Fig. 17b); internal seta flattened but only slightly wider near apex (Fig. 19b)….. *An*. *homunculus* (in part)
- Ventral claspette with a rounded lateral expansion, not forming an anteriorly directed lobe (Fig. 20a); internal seta flattened apically…..*An*. *pholidotus* & *An*. *gonzalezrinconesi*

**Fig. 20:**
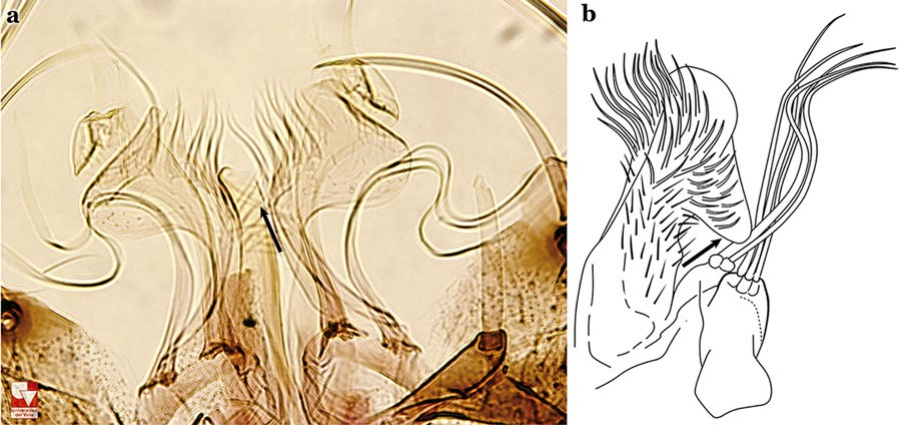
**a***An*. *pholidotus* Zavortink, 1973. **b** *An*. *lepidotus* Zavortink, 1973 (redrawn after Zavortink [13])
- Ventral claspette slightly emarginated and amply expanded laterally, forming a rounded anteriorly directed lobe (Fig. 20b); internal seta not flattened apically…..*An. lepidotus*
- Ventral claspette with a large lateral expansion, curved posteroventrally and forming a sharp anteriorly directed point (Fig. 21a)….. *An*. *homunculus* (in part)

**Fig. 21:**
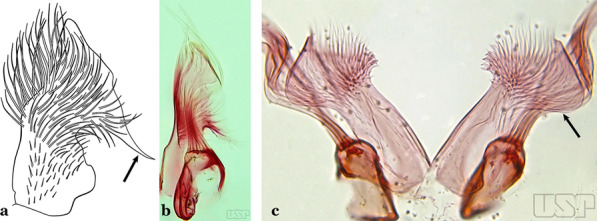
**a**, **b***An*. *homunculus* (**a** redrawn after Zavortink [13]). **c** *An*. *laneanus* Correa & Cerqueira, 1944
- Ventral claspette with a lateral expansion not forming a sharp anteriorly directed point or with a rounded posteriorly directed lobe (Fig. 21b)…..15
- Ventral claspette with a lateral expansion varying from more or less rounded to sinuous on lateral margin, not posteriorly curved (Fig. 22a)….. *An. cruzii* (*s.l*.)

**Fig. 22:**
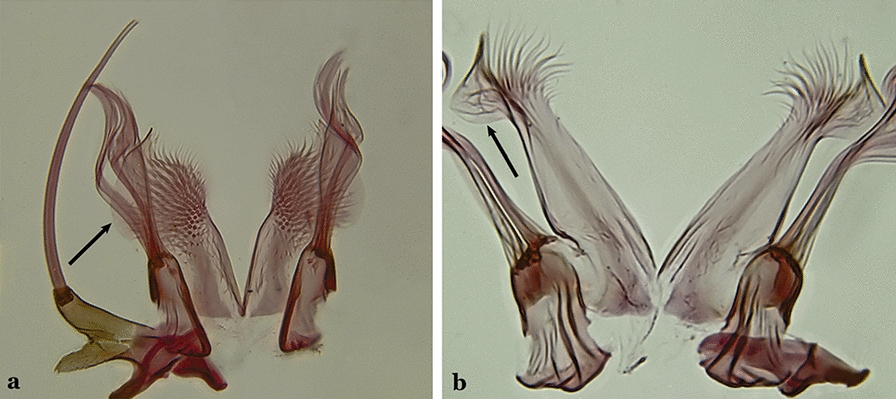
**a***An*. *cruzii*. **b***An*. *bellator* Dyar & Knab, 1906
- Ventral claspette with a rounded lateral expansion, curved posteroventrally, densely spiculate medially (Fig. 22b)…..16
- Ventral claspette spiculose medially, with few short spicules laterally; spiculose portion up to 0.25 length of ventral claspette (Fig. 23a)…..*An. bellator*

**Fig. 23:**
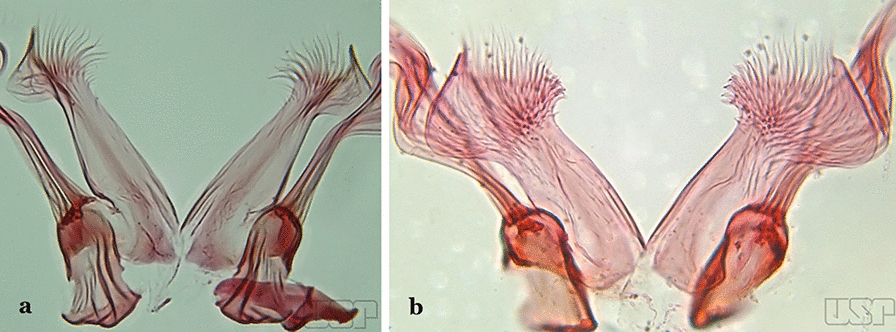
**a***An*. *bellator*. **b***An*. *laneanus*
- Ventral claspette densely spiculose medially, with many short spicules laterally that extend to lateral margin; spiculose portion approximately 0.40 length of ventral claspette (Fig. 23b)…..*An. laneanus*
- Ventral claspette without spicules or setae or with only small spicules mesally on basoventral surface (Fig. 24a)…..18

**Fig. 24:**
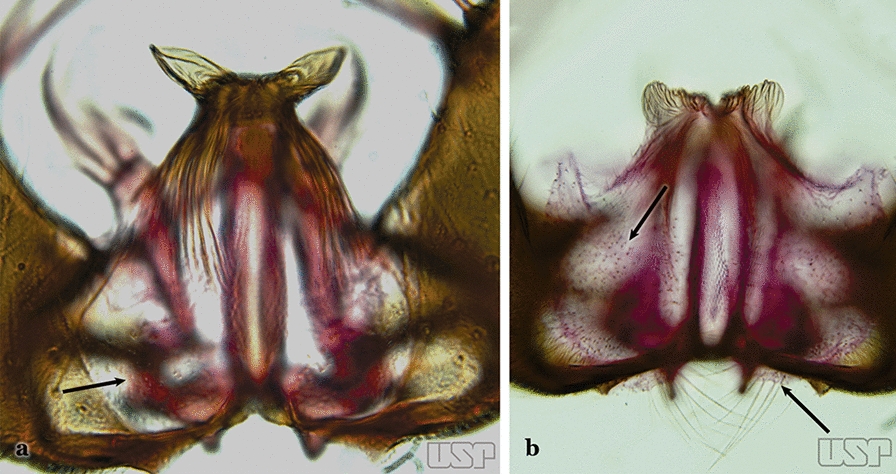
**a***An*. *triannulatus*. **b***An*. *strodei*
- Ventral claspette with spicules or setae at least on basal lobe (Fig. 24b)…..34
- Ventral claspette with a laterally expanded apex, forming a well- developed apicolateral ear-like lobe (Fig. 25a)…..19

**Fig. 25:**
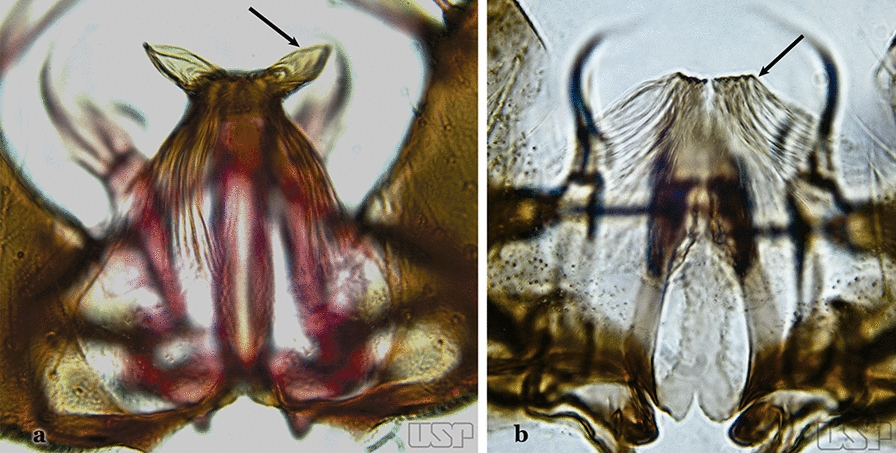
**a***An*. *triannulatus*. **b***An*. *guarani* Shannon, 1928
- Ventral claspette with or without an apicolateral expansion, if expansion present it is never ear-like (Fig. 25b)…..20
- Apicolateral lobes moderately narrow basally and directed laterally or posterolaterally (Fig. 26a)….. *An. triannulatus*

**Fig. 26:**
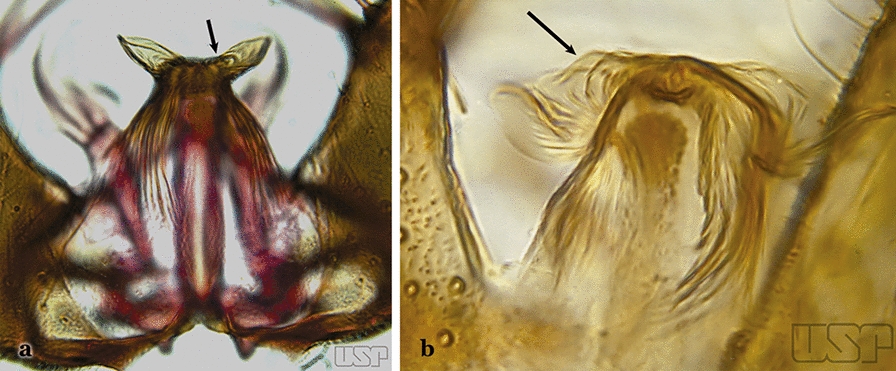
**a***An*. *triannulatus*. **b***An*. *halophylus*
- Apicolateral lobes wide basally and generally directed anteriorly (Fig. 26b)….. *An. halophylus*
- Aedeagus with a pair of well-sclerotized subapical leaflets (Fig. 27a)…..21

**Fig. 27:**
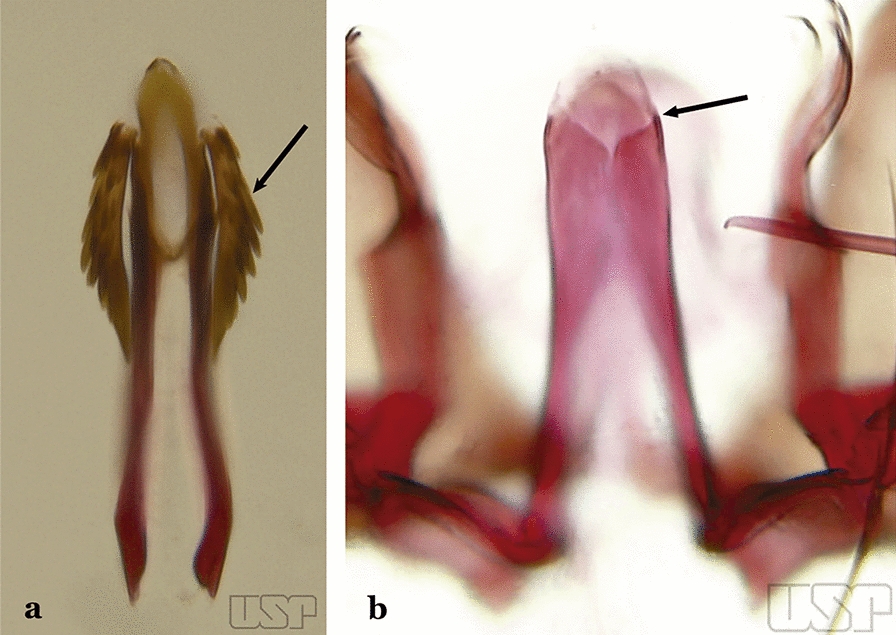
**a***An*. *guarani*. **b***An*. *albitarsis* Lynch Arribálzaga, 1938
- Aedeagus without leaflets (Fig. 27b)…..31
- Apex of aedeagus sclerotized centrally, hyaline laterally, ending in a dorsally curved hook (Fig. 28a)…..*An. parvus*

**Fig. 28:**
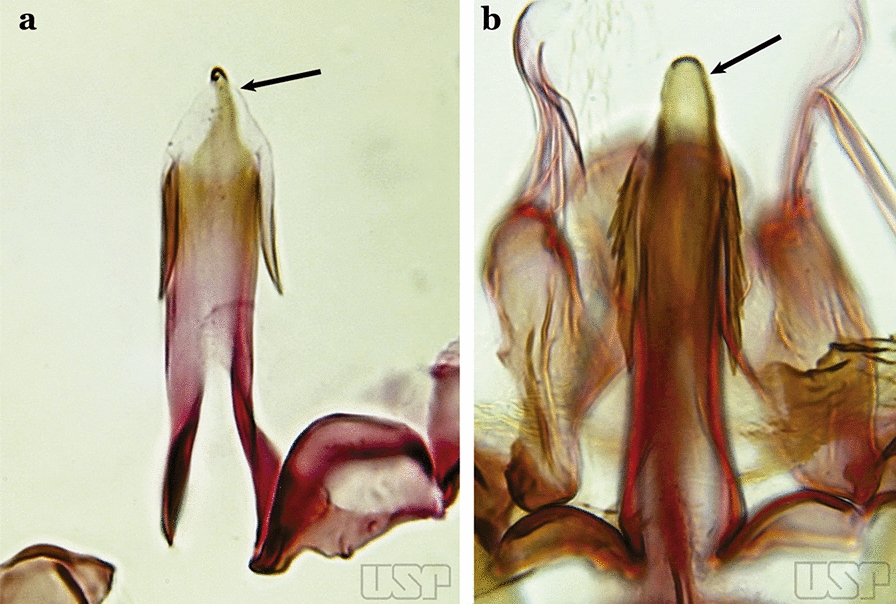
**a***An*. *parvus* (Chagas, 1907). **b** *An*. *antunesi* Galvão & Franco do Amaral, 1940
- Apex of aedeagus not as above, straight, without an apical hook (Fig. 28b)…..22
- Dorsal claspette with 2 apical and 1 subapical setae (Fig. 29a)…..23

**Fig. 29:**
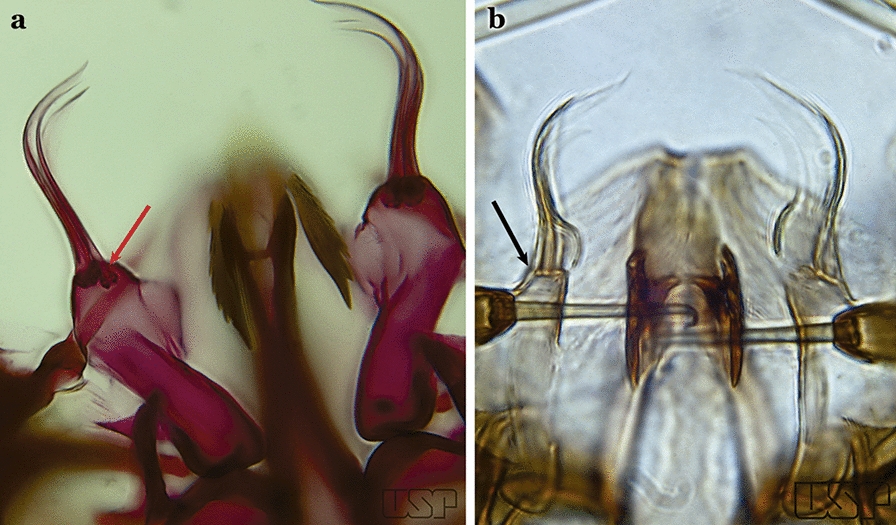
**a***An*. *atacamensis* González & Sallum, 2010. **b** *An*. *darlingi*
- Dorsal claspette with all setae inserted apically (Fig. 29b)…..28
- Proctiger smooth or with minute spines laterally (Fig. 30a)…..24

**Fig. 30:**
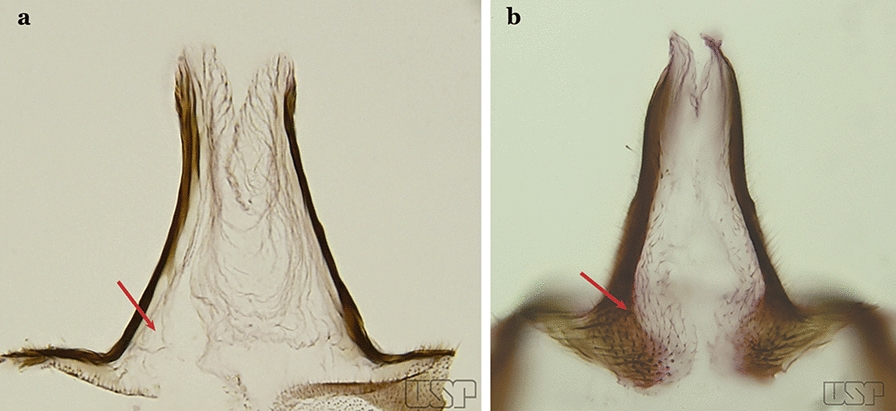
**a***An*. *antunesi*. **b***An*. *atacamensis*
- Proctiger spiculose, at least basally (Fig. 30b)…..25
- Ventral claspette with a narrow apex; aedeagus with subapical leaflets positioned parallel to its longitudinal axis (Fig. 31a)…..*An. antunesi*

**Fig. 31:**
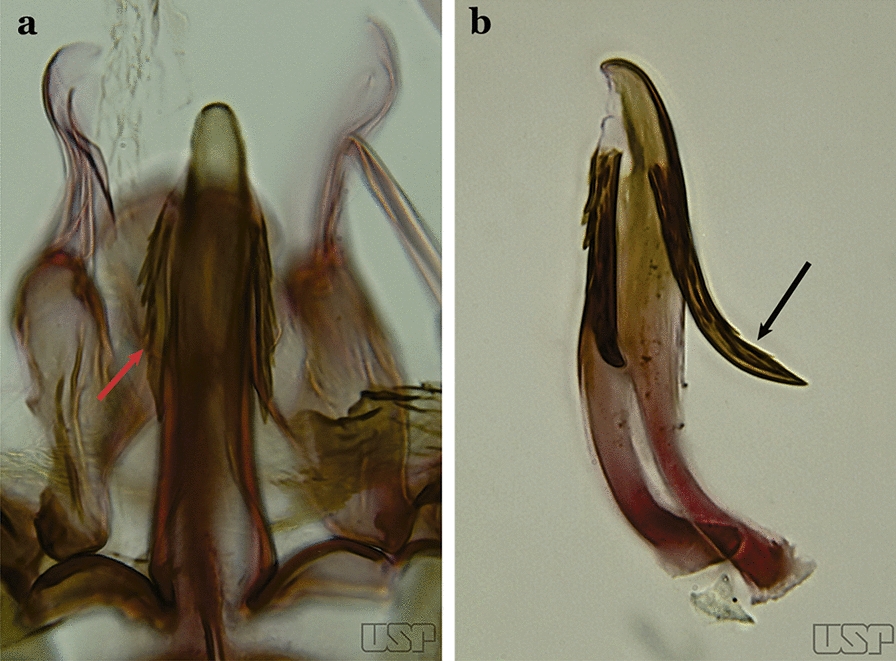
**a***An*. *antunesi*. **b***An*. *pristinus* Nagaki & Sallum, 2010
- Ventral claspette with a rounded apex; aedeagus with subapical leaflets at about a 25° angle in relation to its longitudinal axis (Fig. 31b)….. *An. pristinus*
- Internal seta of gonocoxite straight (Fig. 32a)…..26

**Fig. 32:**
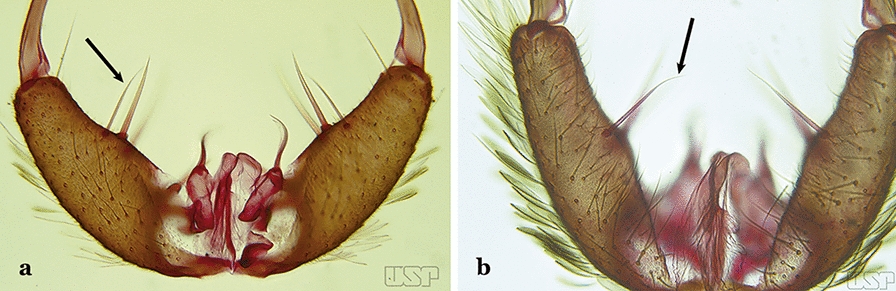
**a***An*. *pictipennis* (Philippi, 1865). **b** *An*. *lutzii* Cruz, 1901
- Internal seta of gonocoxite distally curved (Fig. 32b)…..27
- Aedeagus with a conical or rounded apex; ventromedian subtriangular projection forming a large deep arch (Fig. 33a)…..*An. atacamensis*

**Fig. 33:**
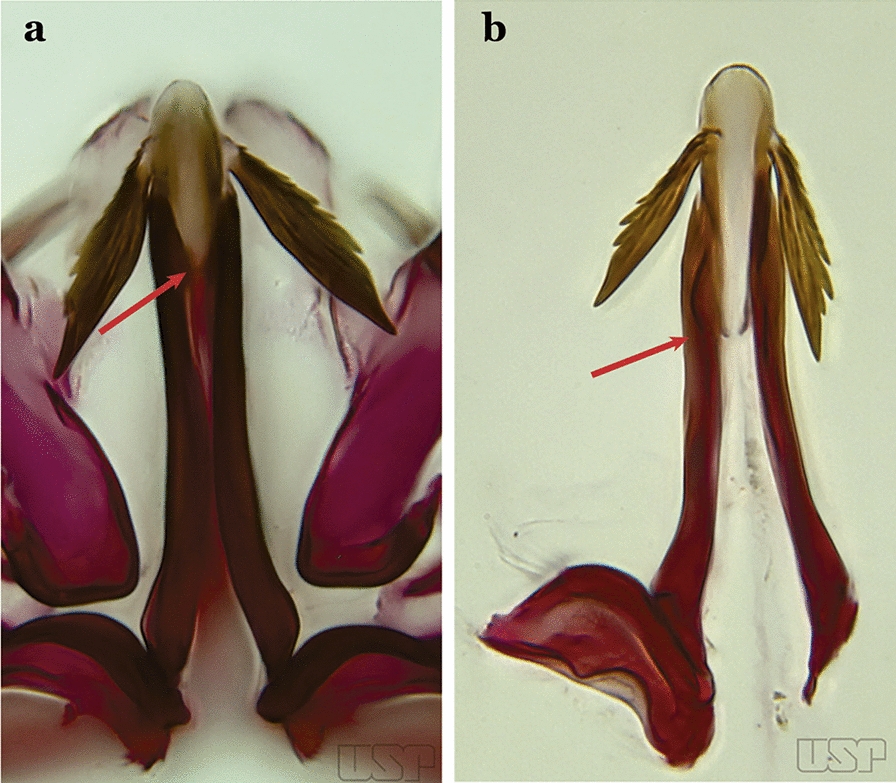
**a***An*. *atacamensis*. **b***An*. *pictipennis*
- Aedeagus with a rounded apex; ventromesal subtriangular projection forming an open narrow arch (Fig. 33b)….. *An. pictipennis*
- Ventral claspette with a large basoventral lobe (Fig. 34a); aedeagus with subapical leaflets directed laterally, forming an angle of about 45° in relation to its longitudinal axis (Fig. 34b)…..*An. lutzii*

**Fig. 34:**
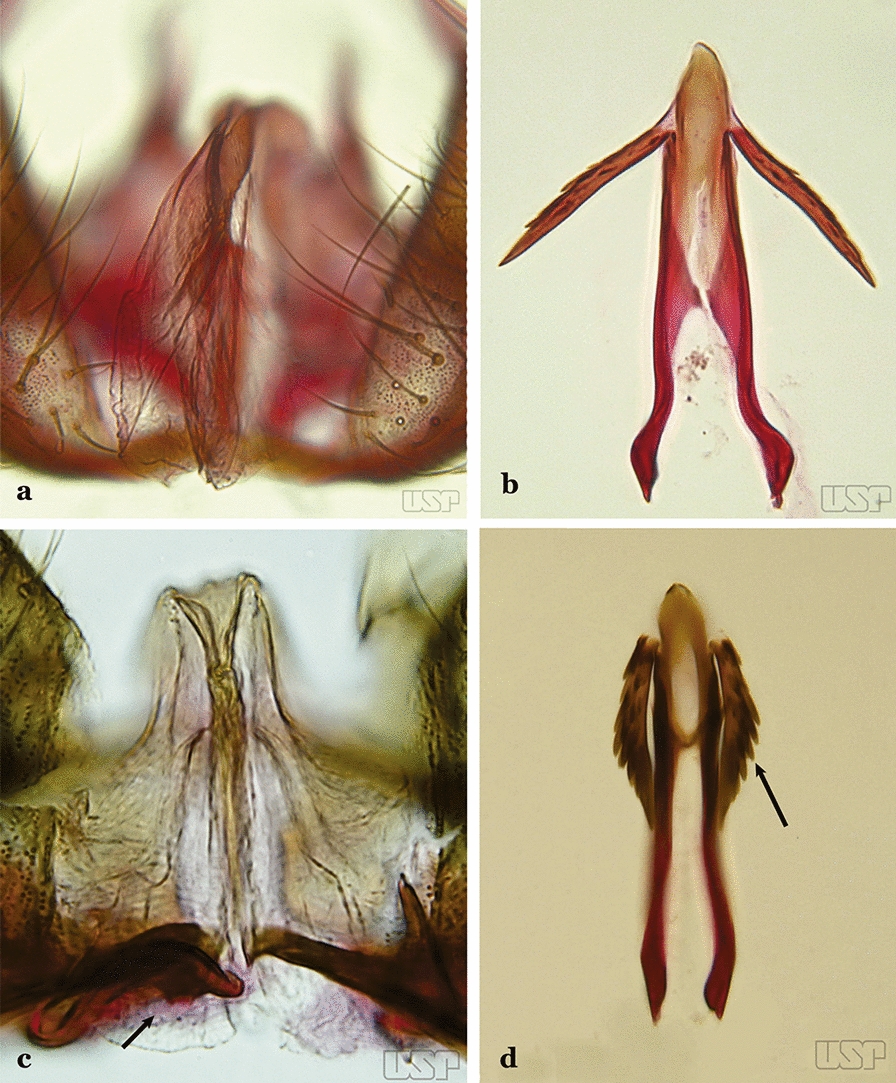
**a**, **b***An*. *lutzii*. **c**, **d***An*. *guarani* Shannon, 1928
- Ventral claspette with a small basoventral lobe (Fig. 34c); aedeagus with subapical leaflets directed posteriorly, positioned approximately parallel to its longitudinal axis (Fig. 34d)….. *An. guarani*
- Apex of aedeagus longer than wide; ventromesal triangular projection of aedeagus envelops all of subapical region, forming a distinct collar (Fig. 35a); dorsal seta of dorsal claspette with a well-defined basomedian projection (Fig. 29b); apex of ventral claspette moderately wide and truncate; median sulcus small, often indistinguishable (Fig. 35b)….. *An. darlingi*

**Fig. 35:**
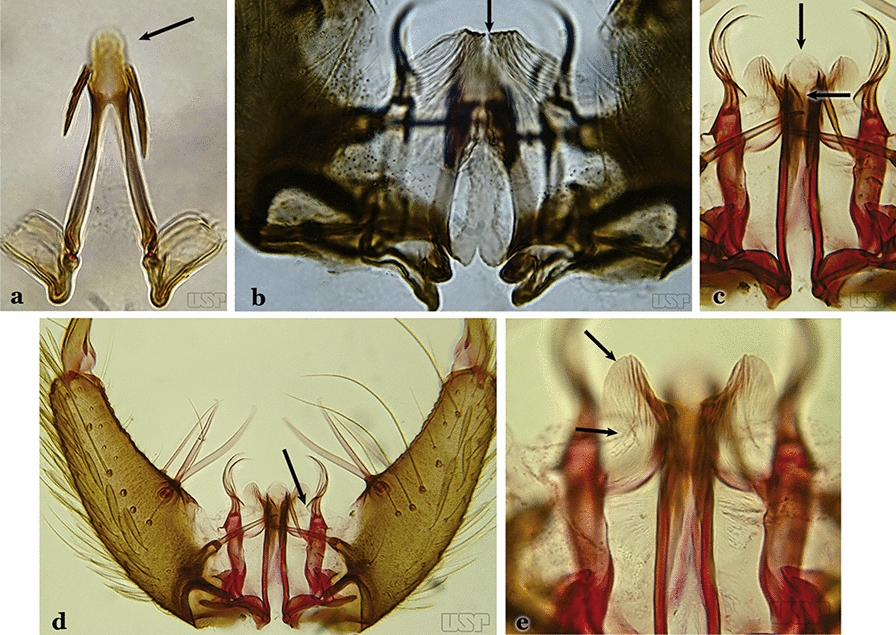
**a**, **b***An*. *darlingi*. **c**, **d**, **e***An*. *lanei* Galvão & Franco do Amaral, 1938
- Apex of aedeagus wider than long (Fig. 35c); ventromesal triangular projection of aedeagus usually absent, when present, it is in form of a collar; dorsal seta of dorsal claspette with or without a well-defined basomedial projection (Fig. 35d); apex of ventral claspette variable; median sulcus large, clearly distinguishable (Fig. 35e)…..29
- Ventral claspette with apex laterally expanded into a large rounded lobe, directed posteriorly, distal third distinctly narrowed (Fig. 35e); aedeagus with relatively long slightly serrated subapical leaflets (Fig. 36a); aedeagus without ventromedial triangular projection; dorsal seta of dorsal claspette with a prominent basomedial projection (Fig. 35d)….. *An. lanei*

**Fig. 36:**
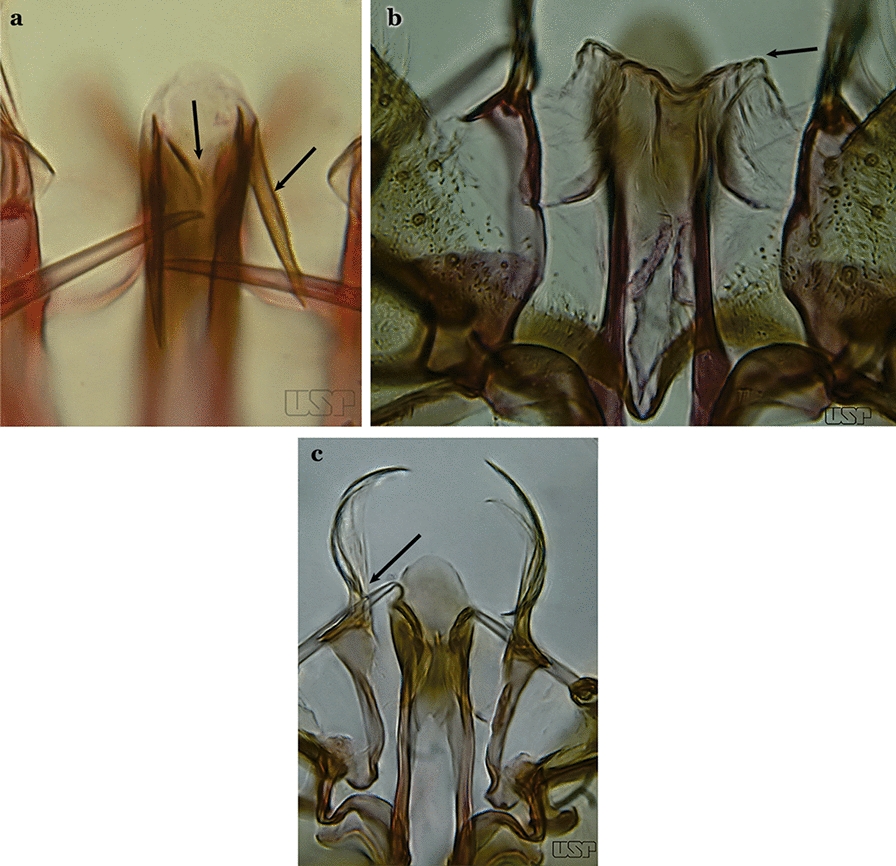
**a***An*. *lanei*. **b**, **c***An*. *argyritarsis*
- Ventral claspette with apex not expanded or slightly expanded laterally, distal third not narrowed (Fig. 36b); aedeagus with strongly serrated subapical leaflets (Fig. 35a); aedeagus with a distinct ventromedial triangular projection (Fig. 35a); dorsal seta of dorsal claspette without a basomedial projection, or not prominent if present (Fig. 36c)…..30
- Ventral claspette with 2 rounded apicolateral expansions; median sulcus wide, distinct, sclerotized medially (Fig. 37a)…..*An. sawyeri*

**Fig. 37:**
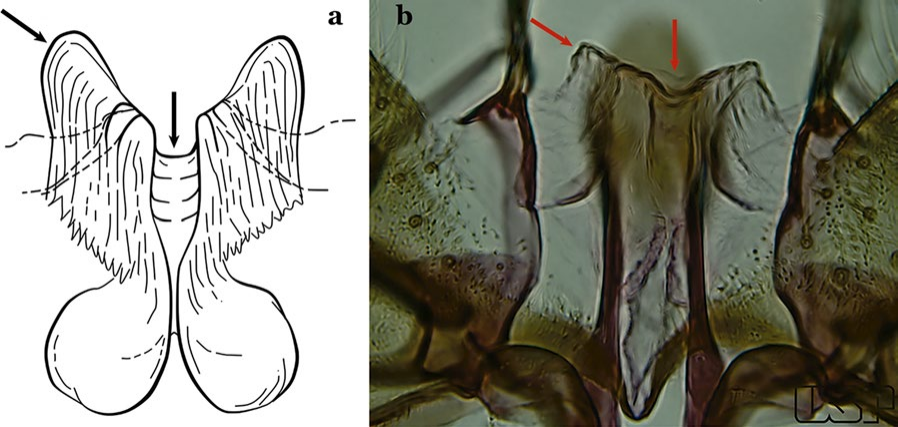
**a***An*. *sawyeri* Causey, Deane, Deane & Sampaio, 1943. **b** *An*. *argyritarsis*
- Ventral claspette without apicolateral expansions; median sulcus narrow, indistinct (Fig. 37b)…..*An. argyritarsis*
- Ventral claspette with a pair of sack-like dilations on its ventral surface, situated just below apex; preapical plate small, well sclerotized (Fig. 38a)…..*An. albimanus*

**Fig. 38:**
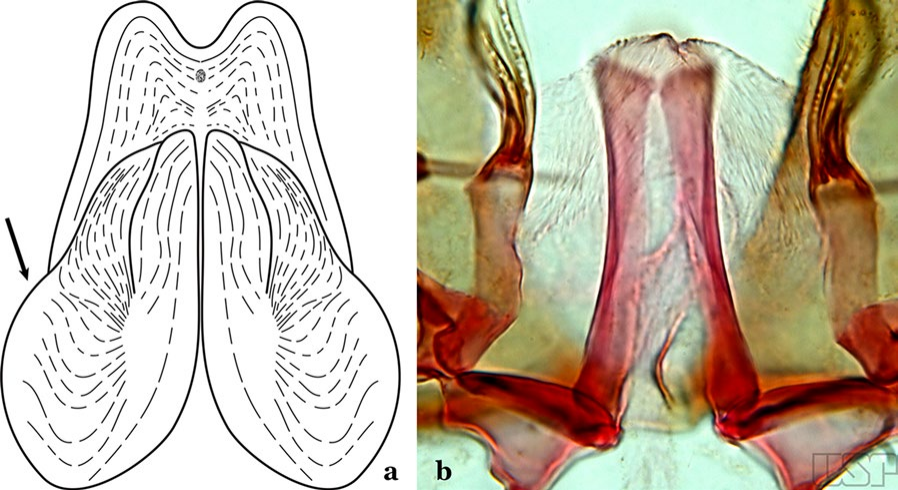
**a***An*. *albimanus* (redrawn after Faran [9]). **b** *An*. *marajoara* Galvão & Damasceno, 1942
- Ventral claspette not as above (Fig. 38b)…..32
- Ventral claspette with a rounded apex, triangular or conical in appearance (Fig. 39a)…..*An. albitarsis*, *An. deaneorum*, *An. janconnae*, *An. marajoara* & *An. oryzalimnetes*

**Fig. 39:**
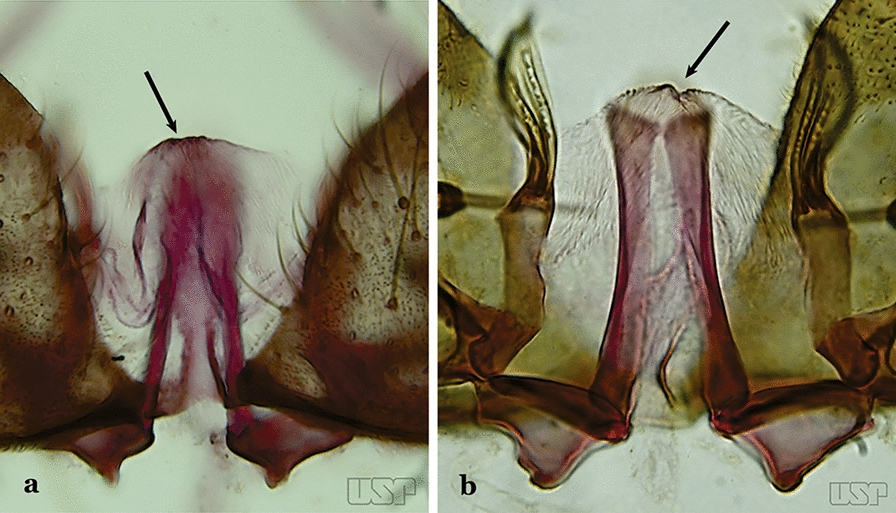
**a***An*. *albitarsis* Lynch Arribálzaga, 1878. **b** *An*. *marajoara*
- Ventral claspette with apex truncate or slightly, rounded trapezoidal in appearance (Fig. 39b)…..33
- Parabasal seta with a hook-like apex, inserted on a well-developed parabasal lobe (Fig. 40a); dorsal seta of dorsal claspette with basomedial projection well-developed; ventral claspette truncate; preapical plate present, distinct (Fig. 40b)…..*An. braziliensis*

**Fig. 40:**
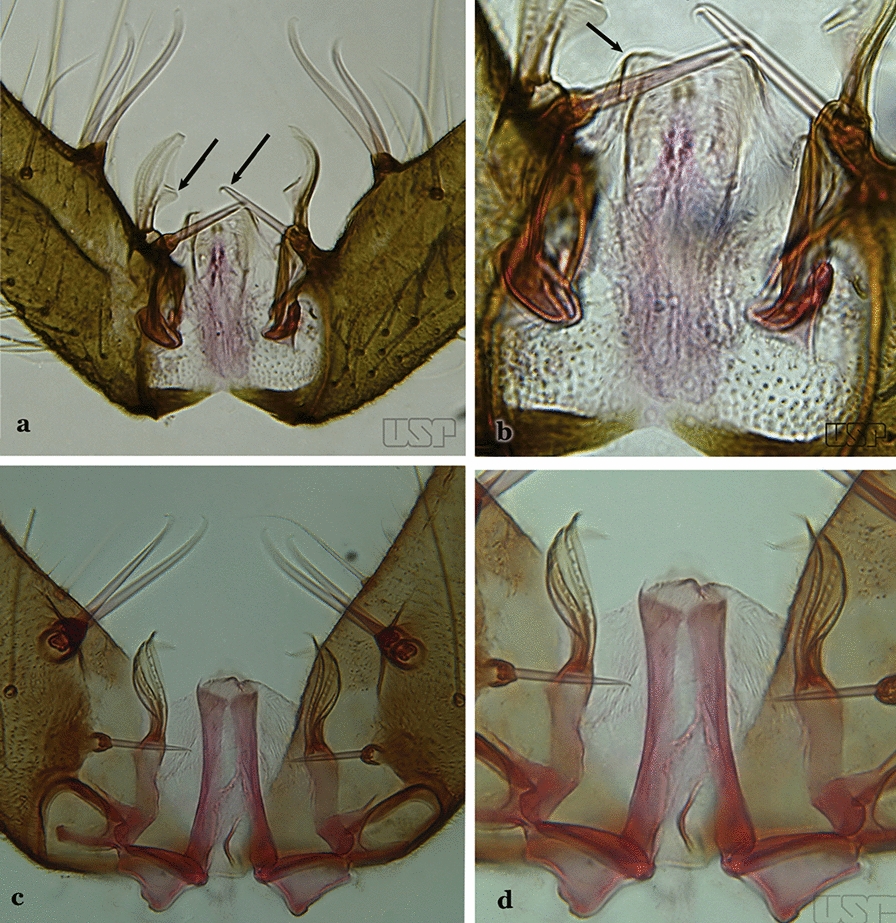
**a**, **b***An*. *braziliensis*. **c**, **d***An*. *marajoara*
- Parabasal seta with a pointed apex, curved, inserted on a reduced parabasal lobe (Fig. 40c); dorsal seta of dorsal claspette without basomedial projection; ventral claspette slightly rounded at apex; preapical plate absent (Fig. 40d)…..*An. marajoara*
- Ventral claspette with a smooth apex, expanded, either rugose or distinctly striate (Fig. 41a)…..35

**Fig. 41:**
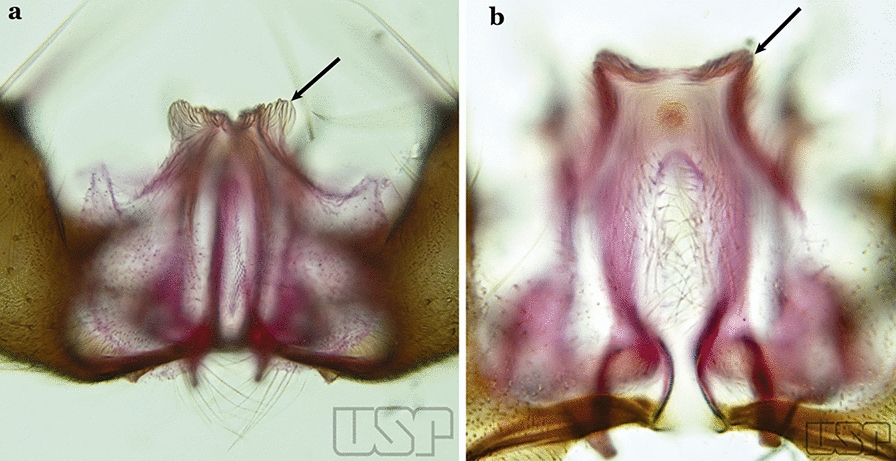
**a***An*. *strodei*. **b***An*. *nuneztovari* Gabaldon, 1940
- Ventral lobe of claspette with a spiculose apex, slightly or not expanded, rugose or striate (Fig. 41b)…..39
- Ventral claspette small, apex moderately expanded laterally, apicolateral margins pointed or moderately angular (Fig. 42a); basoventral lobe of ventral claspette curved in a medial direction (Fig. 42b); preapical plate well sclerotized…..*An. benarrochi*

**Fig. 42:**
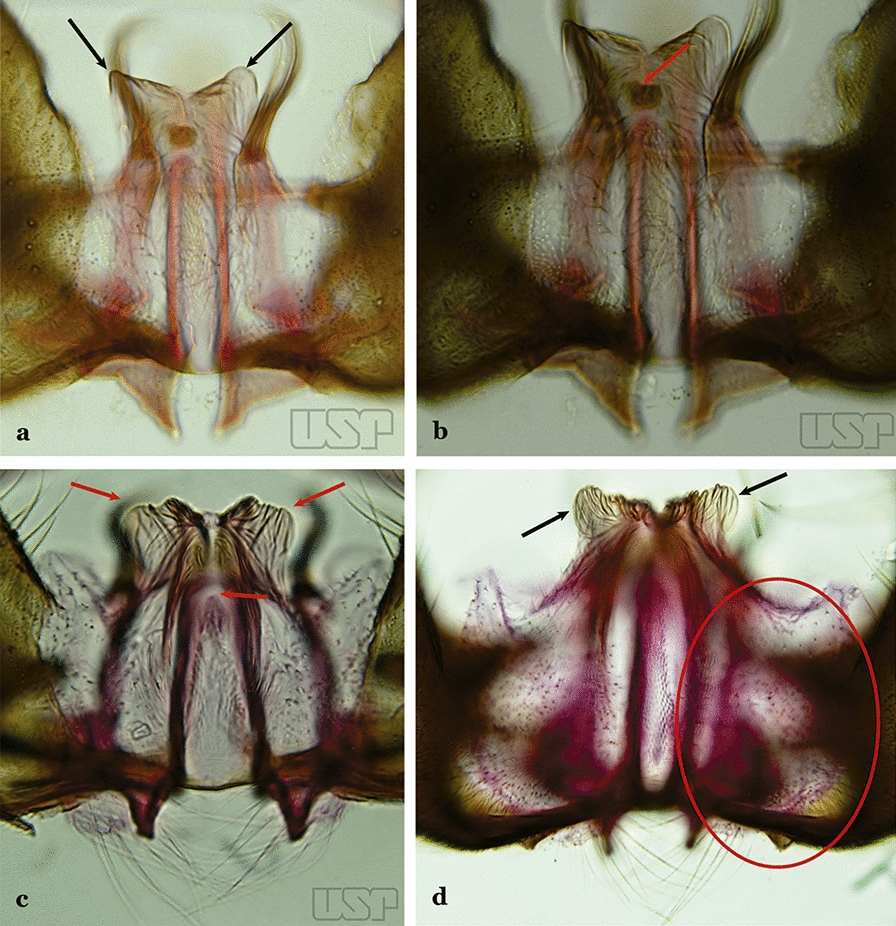
**a**, **b***An*. *benarrochi* Gabaldon, Cova-Garcia & Lopez, 1941. **c** *An*. *rondoni* (Neiva & Pinto, 1922). **d** *An*. *strodei*
- Ventral claspette with apex strongly expanded laterally, apicolateral margins in form of rounded lobes (Fig. 42c, d); basoventral lobe of ventral claspette not as above (Fig. 42d); preapical plate poorly sclerotized or absent (Fig. 42c)…..36
- Ventral claspette with expanded apicolateral margins, quadrangular, lateral margins convex and apical margin slightly concave (Fig. 43a); basoventral lobe large, elongate distally (Fig. 43b); preapical plate poorly developed (Fig. 43a)…..*An. rondoni*

**Fig. 43:**
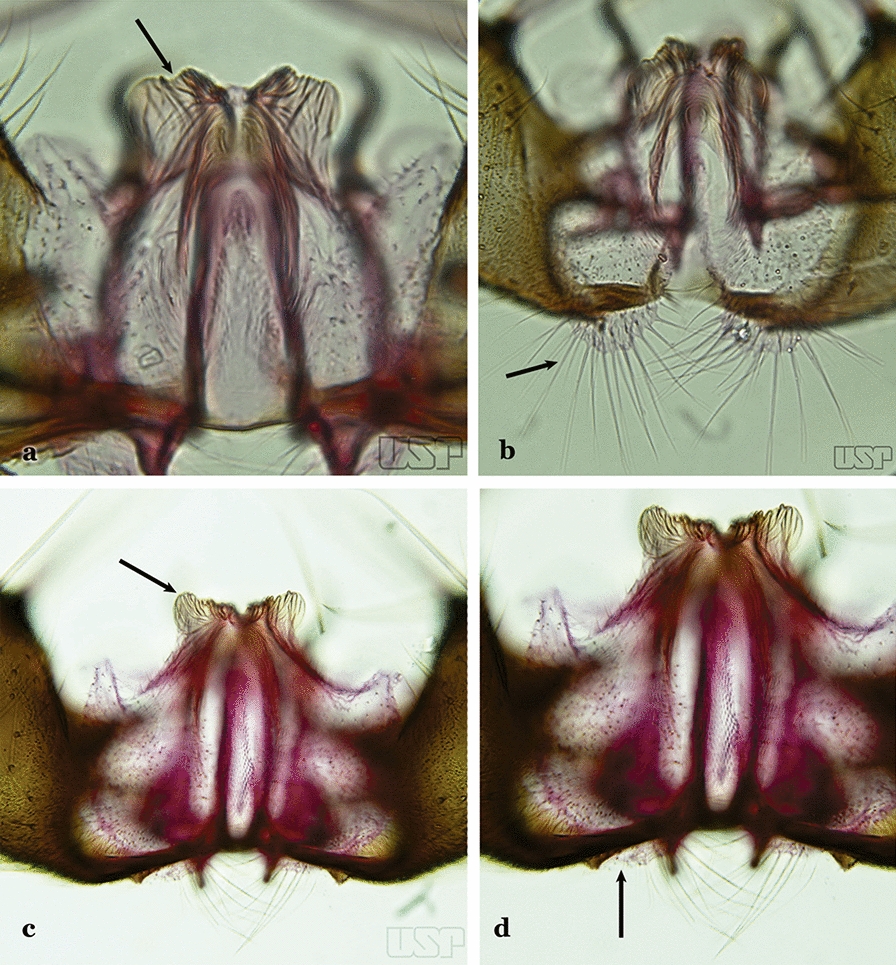
**a**, **b***An*. *rondoni*. **c**, **d***An*. *strodei*
- Ventral claspette with apicolateral margins projected as large rounded lobes, lateral margins convex and apical margin slightly concave (Fig. 43c); basoventral lobe of ventral claspette large, apically rounded (Fig. 43d); preapical plate slightly to moderately defined (Fig. 43c)…..37
- Ventral claspette with apicolateral margins strongly expanded laterally, forming well-developed lobes; ventral claspette without spicules on dorsal and lateral surfaces of apical half (Fig. 44a)….. *An. arthuri* (*s.l*.)

**Fig. 44:**
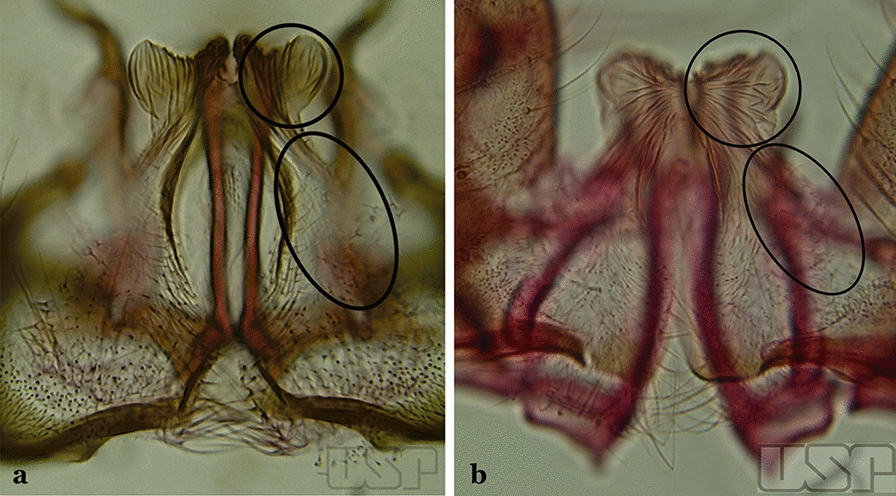
**a***An*. *arthuri* Unti, 1941. **b** *An*. *albertoi* Unti, 1941
- Ventral claspette with apicolateral margins moderately expanded laterally, forming moderately developed lobes; ventral claspette with spicules on dorsal and lateral surfaces of apical half (Fig. 44b)…..38
- Spicules of lateral and dorsal surfaces of apical half of ventral claspette extend to 0.8 of apical portion (Fig. 45a); basoventral lobe of ventral claspette with long well-developed spicules, all similar in size and development, arranged along distal margin…..*An. strodei*

**Fig. 45:**
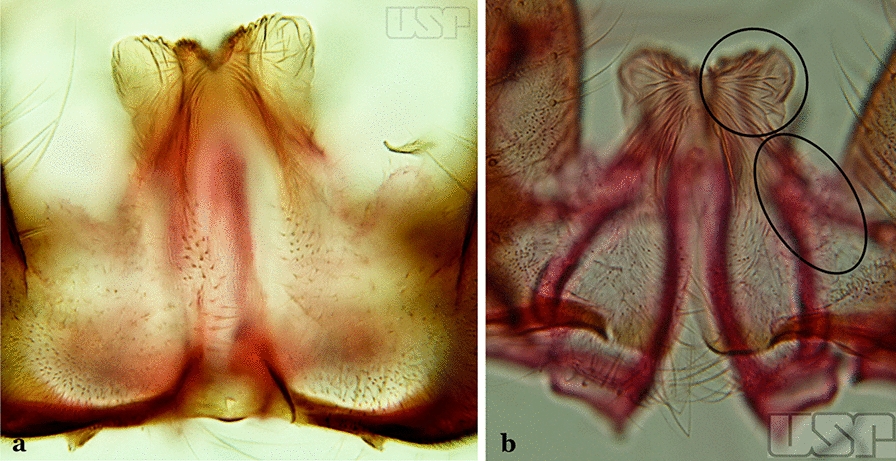
**a***An*. *strodei*. **b***An*. *albertoi*
- Spicules of lateral and dorsal surfaces of apical half of ventral claspette extend to 0.5 of apical portion (Fig. 45b); basoventral lobe of ventral claspette with long and well-developed spicules, spicules denser and longer than on median portion…..*An. albertoi*
- Aedeagus with subapical leaflets (Fig. 46a)…..40

**Fig. 46:**
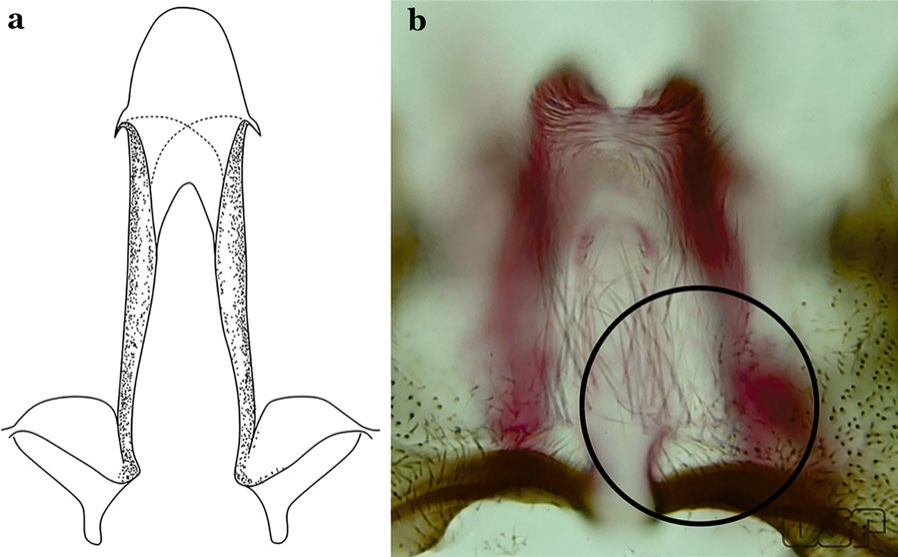
**a***An*. *ininii* (redrawn after Faran [9]). **b** *An*. *goeldii* Rozeboom & Gabaldon, 1941
- Aedeagus without subapical leaflets (Fig. 46b)…..42
- Ventral claspette clearly conical; basal lobe large and curved in a distal direction, with very long spicules; preapical plate large, strongly sclerotized, half- moon-shaped (Fig. 47a); aedeagus with distal extremity membranous, rounded (Fig. 46a)…..*An. ininii*

**Fig. 47:**
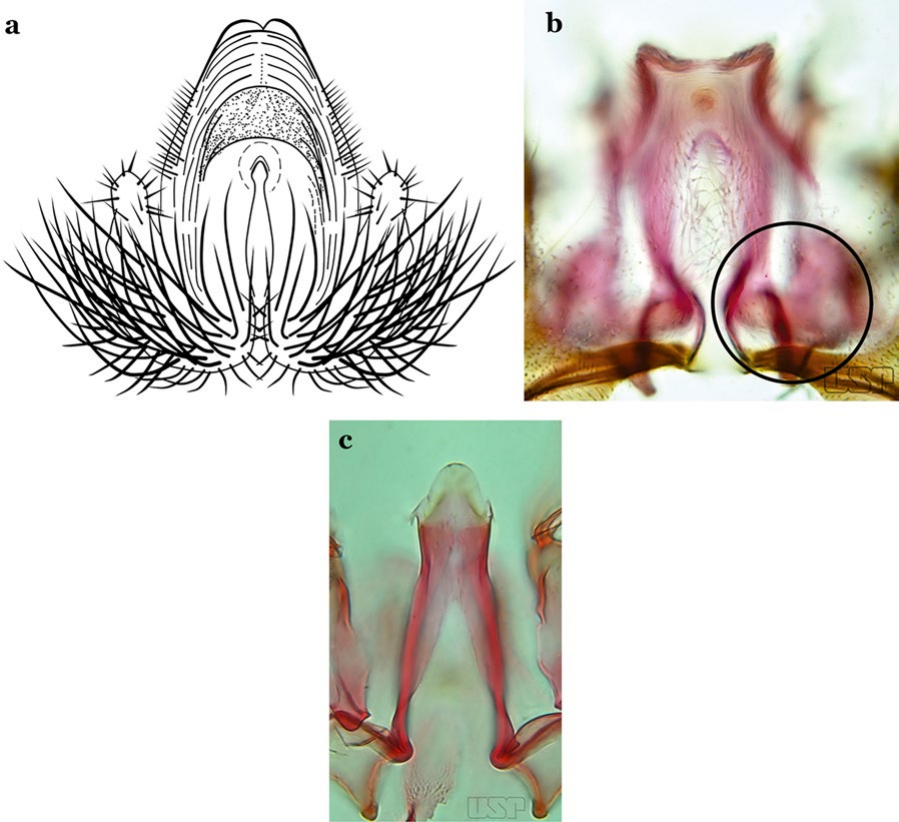
**a***An*. *ininii* Senevet & Abonnenc, 1938 (redrawn after Faran [9]). **b**, **c** *An*. *nuneztovari*
- Ventral claspette rectangular (Fig. 47b); basoventral lobe of ventral claspette rectangular, small, straight, with short spicules (Fig. 46b); preapical plate indistinct (Figs. 46b, 47b); aedeagus with apical extremity membranous, triangular, frequently with small subapical leaflets, more easily visible when aedeagus is separated from other structures of genitalia (Figs. 47c)…..41
- Apex of aedeagus more or less triangular (Fig. 47c); basomedian portion of ventral claspette with short sparse spicules (Fig. 47b)…..*An. nuneztovari*
- Apex of aedeagus more or less quadrangular (Fig. 48); basomedian portion of ventral claspette with moderately long and dense spicules (Fig. 46b)….. *An. goeldii*

**Fig. 48:**
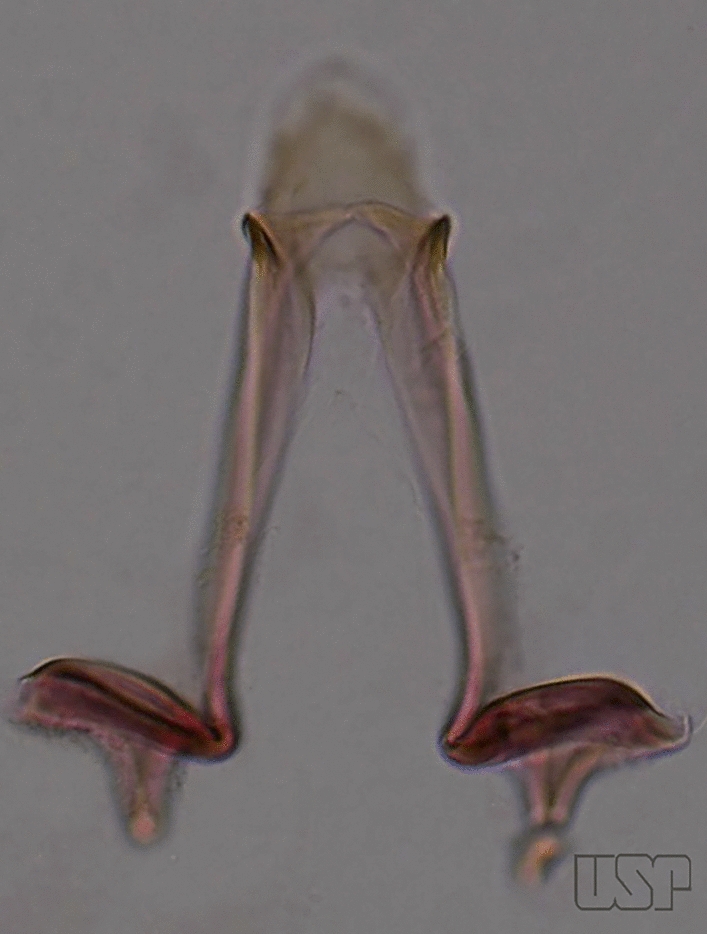
*An*. *goeldii*
- Ventral claspette with a dense array of spicules on basomedian margin of basoventral lobe; preapical plate small and strongly sclerotized (Fig. 49a)….. *An. rangeli*

**Fig. 49:**
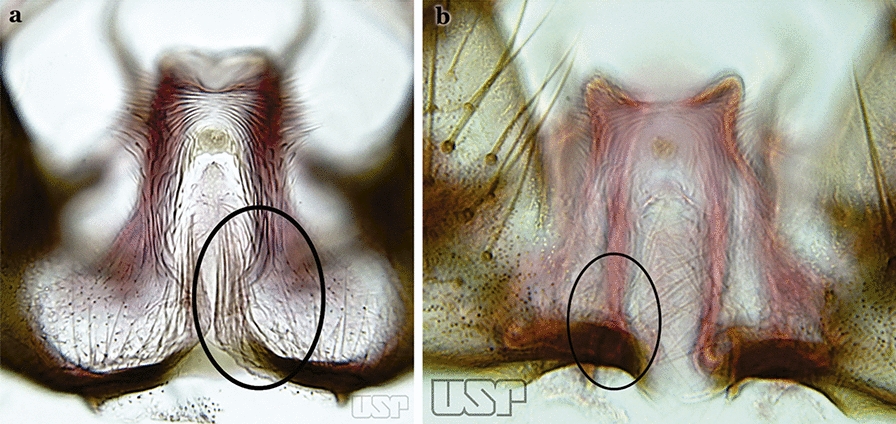
**a***An*. *rangeli* Gabaldon, Cova-Garcia & Lopez, 1940. **b** *An*. *dunhami* Causey, 1945
- Ventral claspette without a dense array of spicules on basoventral lobe; preapical plate not as above (Fig. 49b)…..43
- Ventral claspette with apex broad, quadrangular in outline, forming an angle of about 90° with lateral margin (Fig. 50a)…..*An. dunhami* & *An. trinkae*

**Fig. 50:**
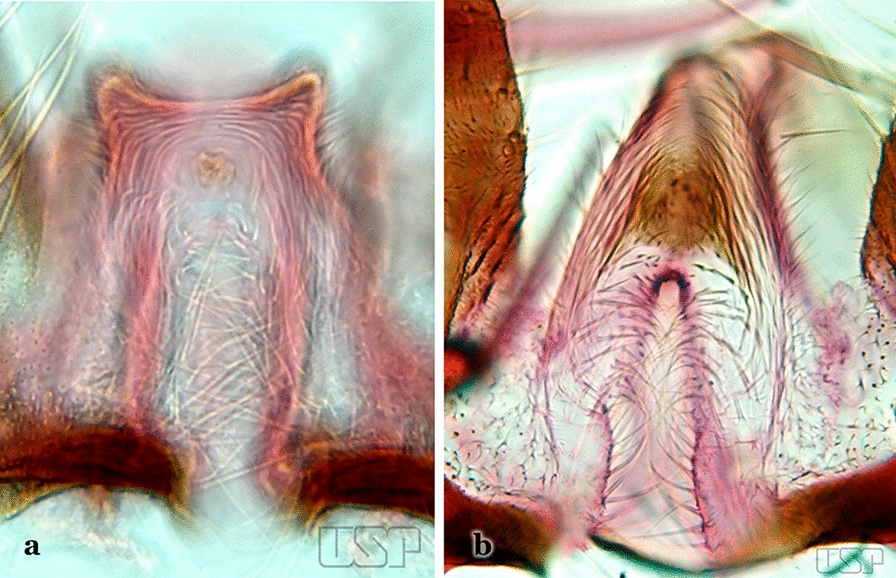
**a***An*. *dunhami*. **b***An*. *galvaoi* Causey, Deane & Deane, 1943
- Ventral claspette with apex narrow, trapezoidal in outline, gradually continuous with lateral margin (Fig. 50b)…..44
- Aedeagus with apical portion very short and truncate (Fig. 51a)…..*An. evansae*

**Fig. 51:**
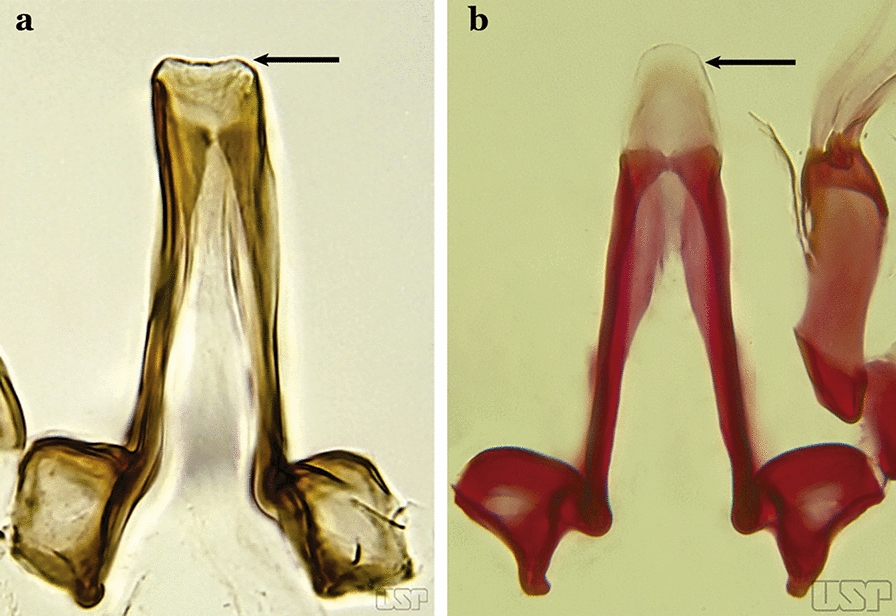
**a***An*. *evansae* (Brèthes, 1926). **b** *An*. *galvaoi*
- Aedeagus with apical portion elongate and rounded (Fig. 51b)…..45
- Ventral claspette with basoventral lobe rounded, with sparse, moderately long spicules on basal margin; spicules of ventral claspette not extending to apex; preapical plate rounded, moderately sclerotized (Fig. 52a)…..*An. aquasalis*

**Fig. 52:**
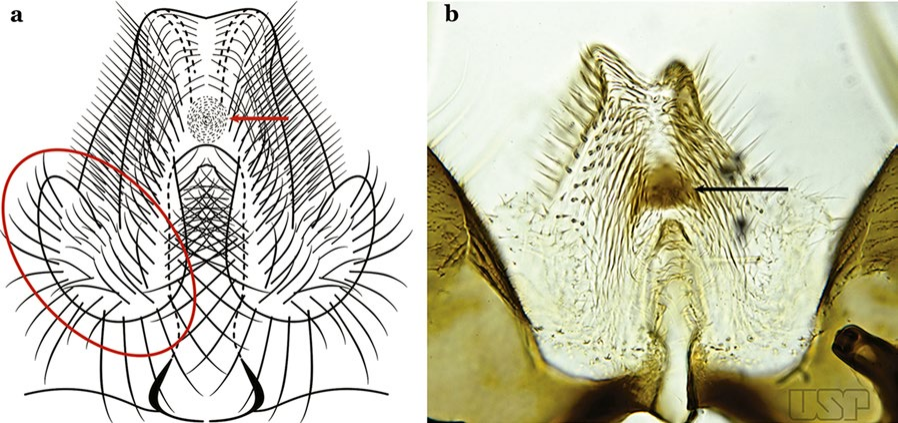
**a***An*. *aquasalis* Curry, 1932 (redrawn after Faran [9]). **b** *An*. *konderi* Galvão & Damasceno, 1942
- Ventral claspette with basoventral lobe rounded, with numerous long spicules on basal margin; spicules of ventral claspette extending to apex; preapical plate half- moon-shaped, strongly sclerotized (Fig. 52b)…..46
- Ventral claspette with long and numerous spicules on basoventral margin of basal lobe, spicule length up to nearly 3 times width of aedeagus (Fig. 53a); preapical plate large, sclerotized (Fig. 53b)….. *An. galvaoi*

**Fig. 53:**
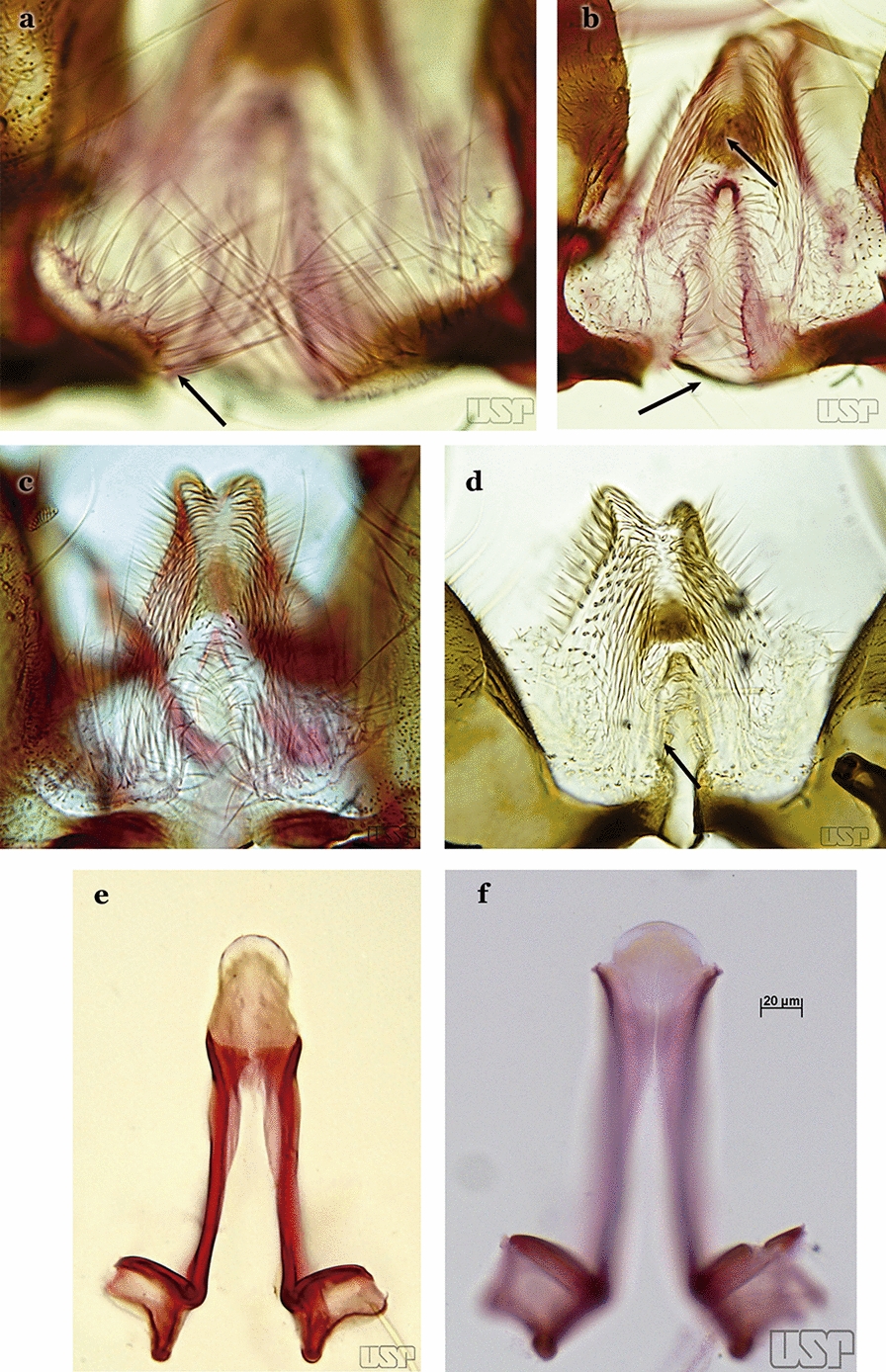
**a**, **b***An*. *galvaoi*. **c, e***An*. *oswaldoi* (Peryassú, 1922). **d, f** *An*. *konderi*
- Ventral claspette with shorter spicules on basal margin of basoventral lobe, spicule length about 2 times width of aedeagus (Figs. 53c, d); preapical plate large, less sclerotized than described above (Figs. 53e, f)…..47
- Apex of aedeagus longer than wide, without subapical points (“leaflets”) (Fig. 53e)…..*An. oswaldoi* (*s.l*.)
- Apex of aedeagus wider than long, with small subapical acute lateral points (“leaflets”) (Fig. 53f)…..*An. konderi* (*s.l*.)
- Aedeagus with 2 pairs of apical leaflets, but only 1 obvious pair (Fig. 54a)…..*An. squamifemur*

**Fig. 54:**
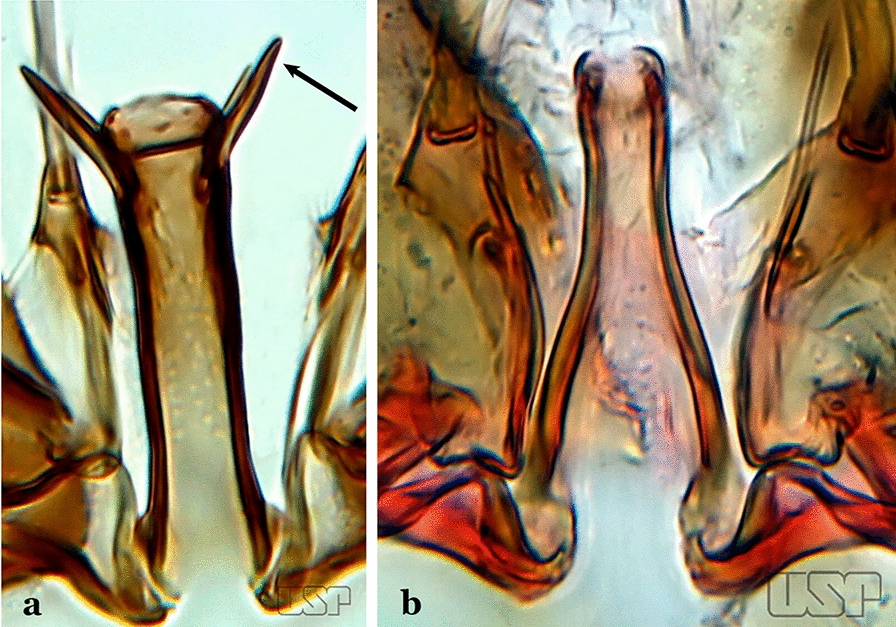
**a***An*. *squamifemur* Antunes, 1937. **b** *An*. *pseudotibiamaculatus* Galvão & Barretto, 1941
- Aedeagus without apical leaflets (Fig. 54b)…..49
- Apical seta of dorsal claspette expanded, spoon-like, with apex rounded (Fig. 55a)…..50

**Fig. 55:**
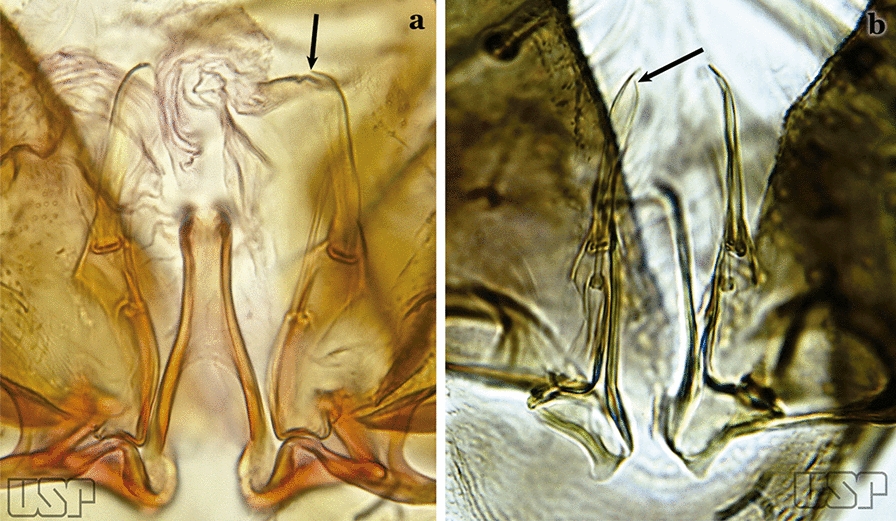
**a***An*. *pseudotibiamaculatus*. **b***An*. *gilesi* (Neiva, 1908)
- Apical seta of dorsal claspette expanded, with apex narrow or lanceolate (Fig. 55b) ………..51
- Aedeagus short, curved and conical, well sclerotized, with apex not expanded (Fig. 56a)…..*An. pseudotibiamaculatus*

**Fig. 56:**
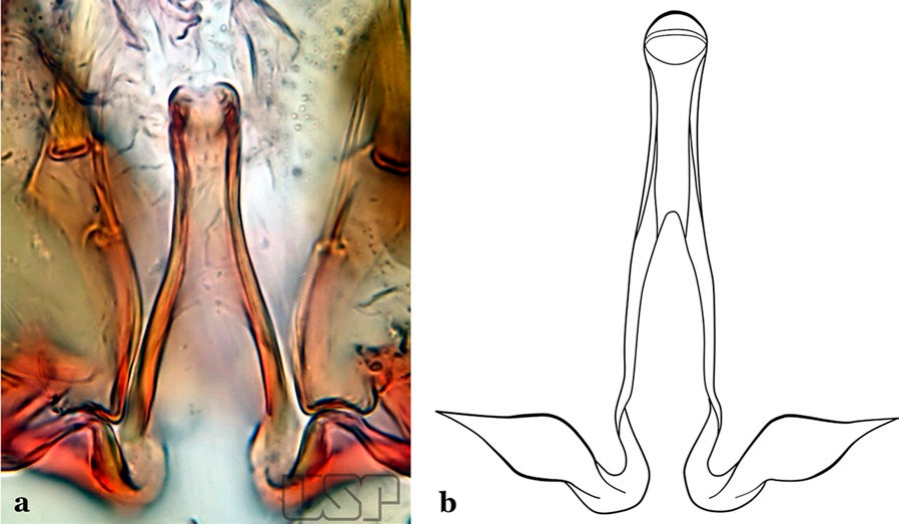
**a***An*. *pseudotibiamaculatus*. **b***An*. *gomezdelatorrei* Levi-Castillo, 1955
- Aedeagus elongated and tubular, with apex expanded and rounded (Fig. 56b)…..*An. gomezdelatorrei*
- Aedeagus hyaline, with apical extremity expanded, semicircular in outline (Fig. 57a)…..*An. vargasi*

**Fig. 57:**
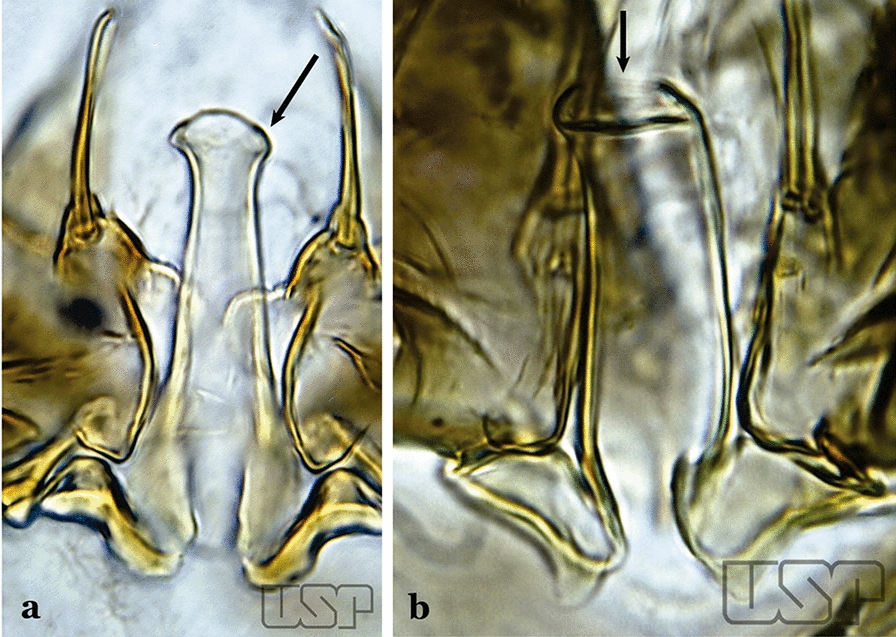
**a***An*. *vargasi* Gabaldon, Cova-Garcia & Lopez, 1941 (redrawn after Levi-Castillo [15]). **b** *An*. *gilesi* (Neiva, 1908)
- Aedeagus not hyaline, with apical extremity not expanded (Fig. 57b)…..52
- Aedeagus with apical extremity quadrangular in outline (Fig. 58a); strongest seta of dorsal claspette single, not branched (Fig. 58b)….. *An. gilesi*

**Fig. 58:**
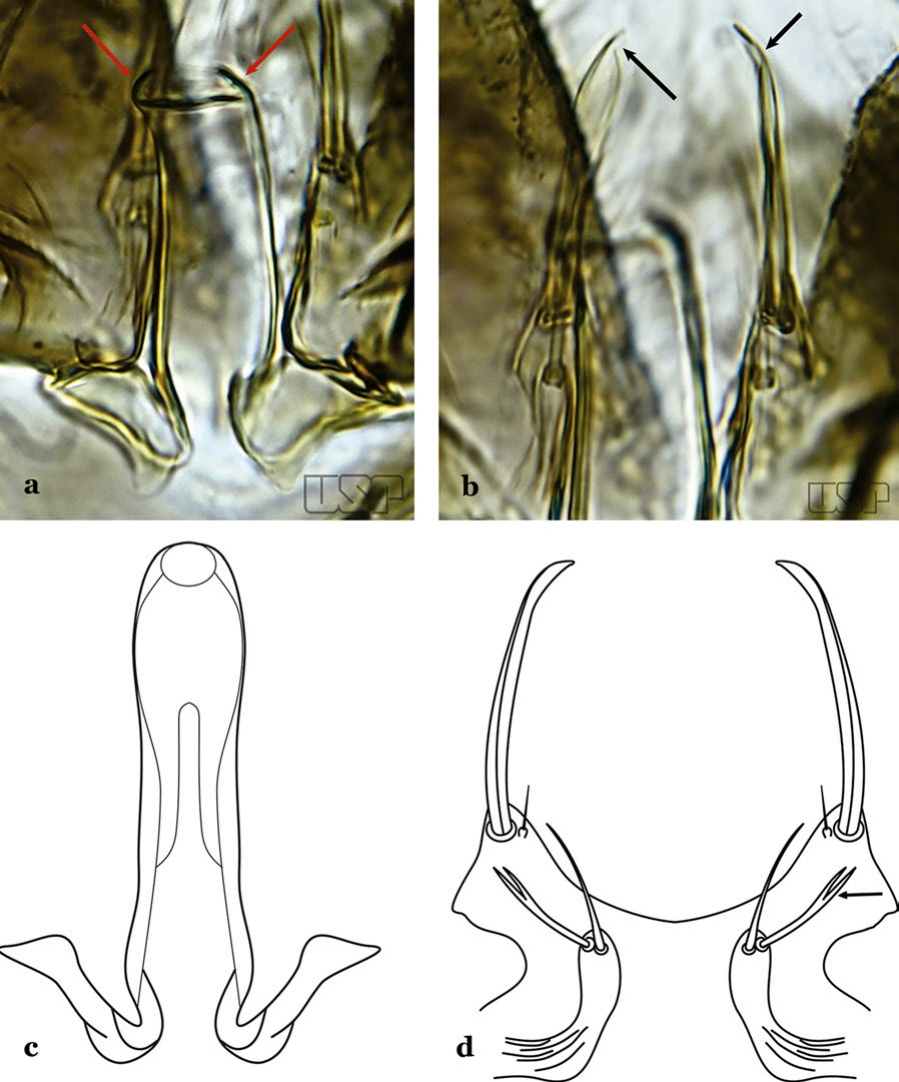
**a**, **b***An*. *gilesi*. **c**, **d***An*. *oiketorakras* Osorno-Mesa, 1947 redrawn after Lane [16]
- Aedeagus with apical extremity triangular or trapezoidal in outline (Fig. 58c); strongest seta of dorsal claspette forked (Fig. 58d)…..*An. oiketorakras*
- Aedeagus without apical leaflets, quadrangular in outline, with 2 lateral rod-like sclerotizations connected basally by a hyaline bridge and basally with 2 lateral hyaline digitiform expansions (Fig. 59a)….. *An. tibiamaculatus*

**Fig. 59:**
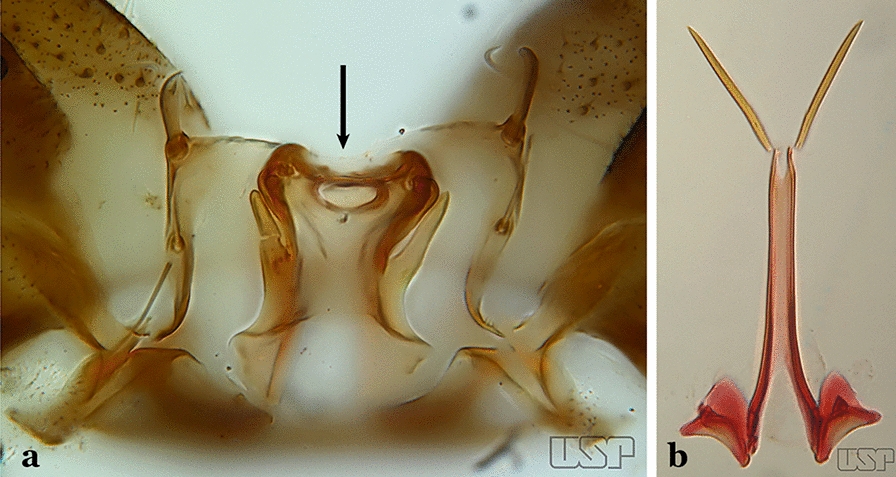
**a***An*. *tibiamaculatus* Neiva, 1906. **b** *An*. *costai*
- Aedeagus with apical leaflets of variable appearance (Fig. 59b)…..54
- Lobes of tergum IX large, nearly parallel, except apically, approximated medially, space between them narrow (Fig. 60a)…..55

**Fig. 60:**
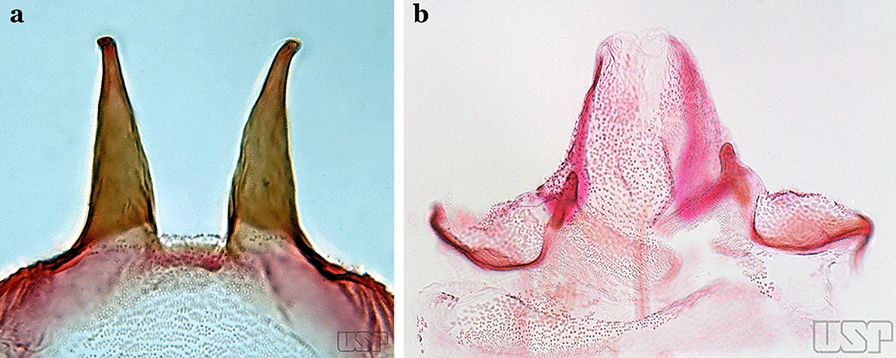
**a***An*. *mediopunctatus* (Lutz, 1903). **b** *An*. *eiseni* Coquillett, 1902
- Lobes of tergum IX small, widely separated, space between them large (Fig. 60b)…..57
- Ventral claspette nearly as long as wide, with a rounded apex in lateral view, without apicodorsal projection, dorsomedial surface with strong and numerous long spicules (Fig. 61a); lobes of tergum IX with a broad base, narrowing toward apex, lobes moderately divergent (Fig. 61b)…..*An. mediopunctatus*

**Fig. 61:**
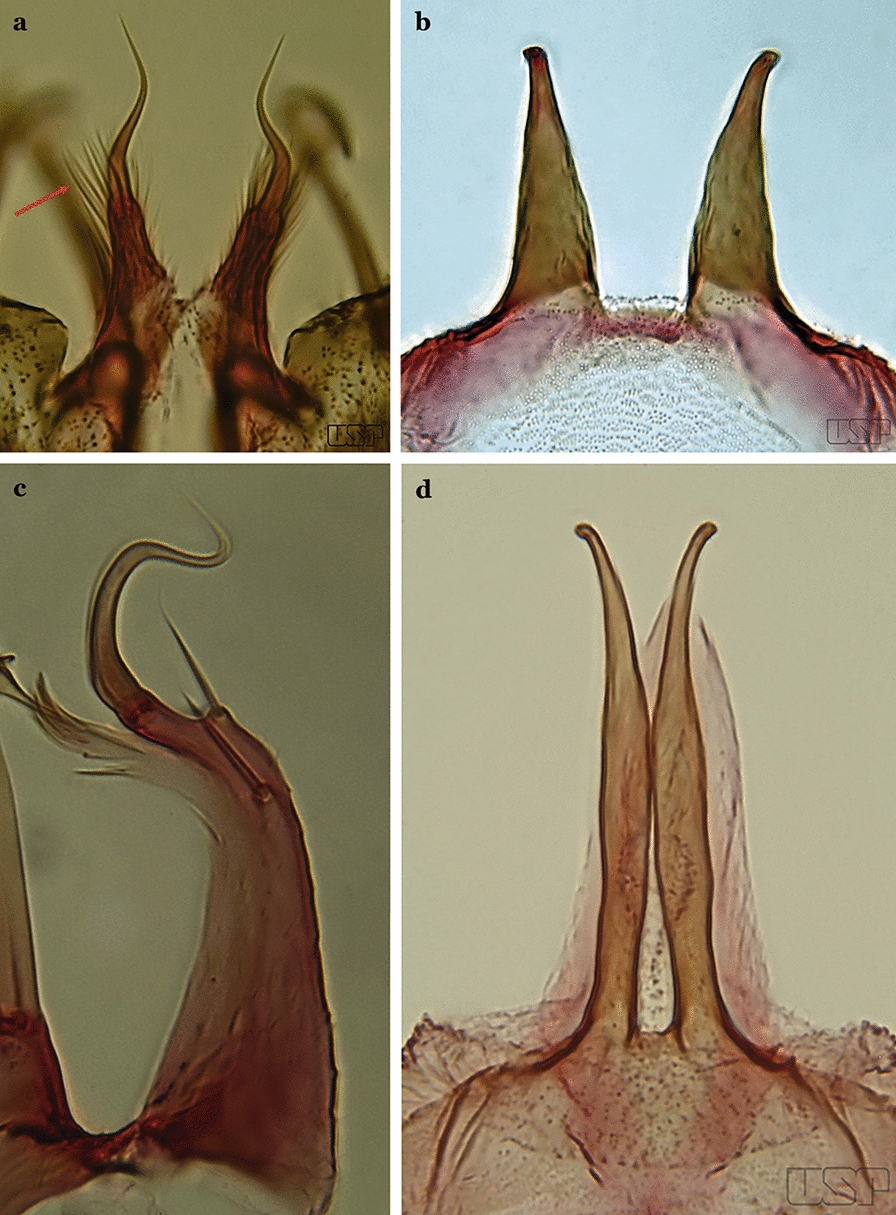
**a**, **b***An*. *mediopunctatus*. **c***An*. *costai*. **d***An*. *forattinii* Wilkerson & Sallum, 1999
- Ventral claspette columnar, longer than wide, apex with apicodorsal projections directed posteriorly, dorsomedial surface with small sparse spicules (Fig. 61c); lobes of tergum IX very close together, parallel, divergent in apical 0.3 (Fig. 61d)…..56
- Dorsal claspette ovoid, almost circular (Fig. 62a); area between dorsal and ventral claspette U-shaped (Fig. 61d)…..*An. costai*

**Fig. 62:**
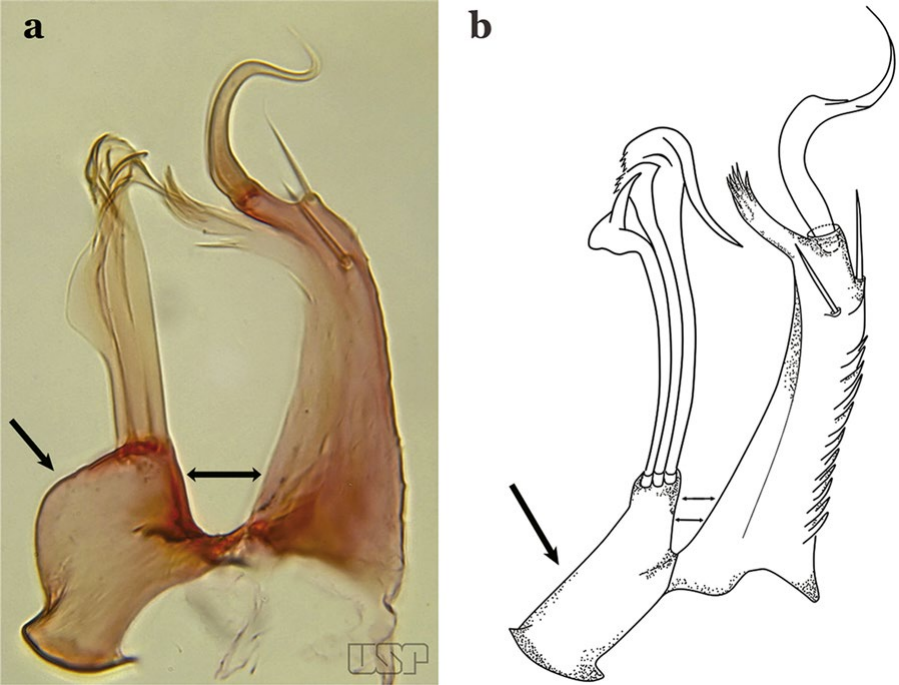
**a***An*. *costai*. **b***An*. *forattinii* redrawn after Wilkerson & Sallum [17]
- Dorsal claspette nearly triangular, never ovoid; area between dorsal and ventral claspette V-shaped (Fig. 62b)…..*An. forattinii*
- Aedeagus with a single pair of leaflets, with smooth or serrate margins (Fig. 63a)…..58

**Fig. 63:**
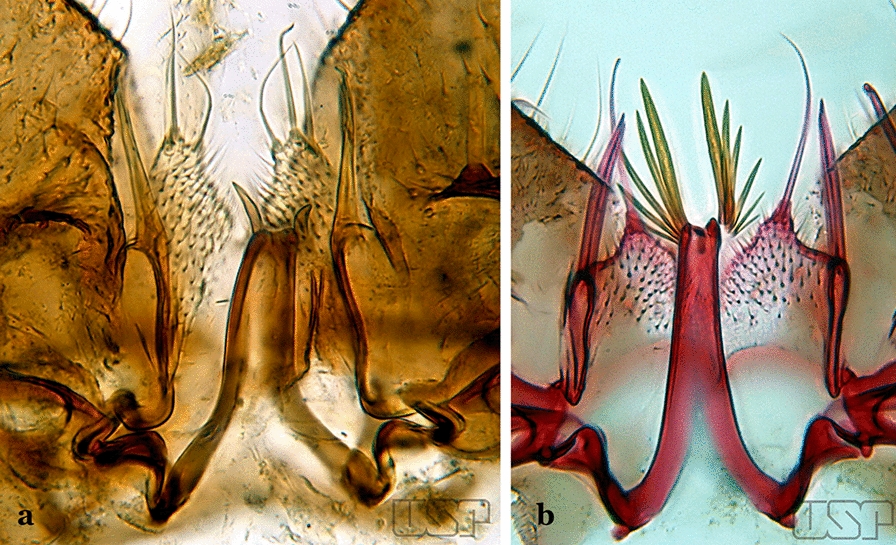
**a***An*. *mattogrossensis* Lutz & Neiva, 191163. **b** *An*. *peryassui*
- Aedeagus with more than one pair of leaflets, with smooth or serrate margins (Fig. 63b)…..60
- Aedeagus with long leaflets, 0.50 to 0.75 length of aedeagus; ventral claspette spiculose, with 1 stout apical seta and 1 or 2 smaller accessory setae (Fig. 64a); dorsal claspette conical, with 2 or 3 apically spatulate, closely approximated, club-like setae (Fig. 64b)…..*An. vestitipennis*

**Fig. 64:**
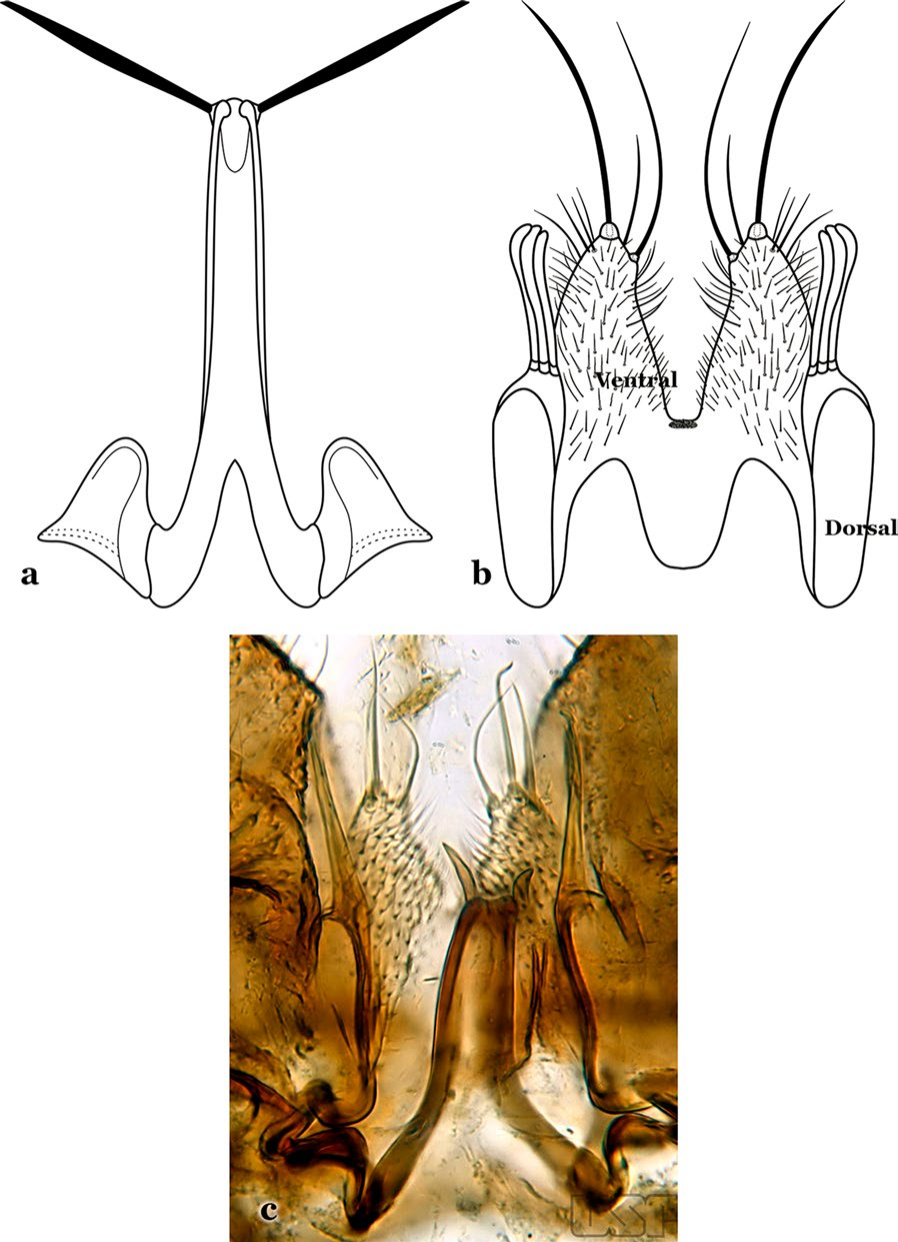
**a**, **b***An*. *vestitipennis* Dyar & Knab, 1906 (redrawn after Komp [7]). **c** *An*. *mattogrossensis* Lutz & Neiva, 1911
- Aedeagus with short leaflets, shorter than 0.5 length of aedeagus (Fig. 64c)…..59
- Aedeagus with strongly sclerotized leaflets (Fig. 64c), with smooth margins; dorsal claspette with setae similar in development; ventral claspette as in Fig. 65a…..*An. mattogrossensis*

**Fig. 65:**
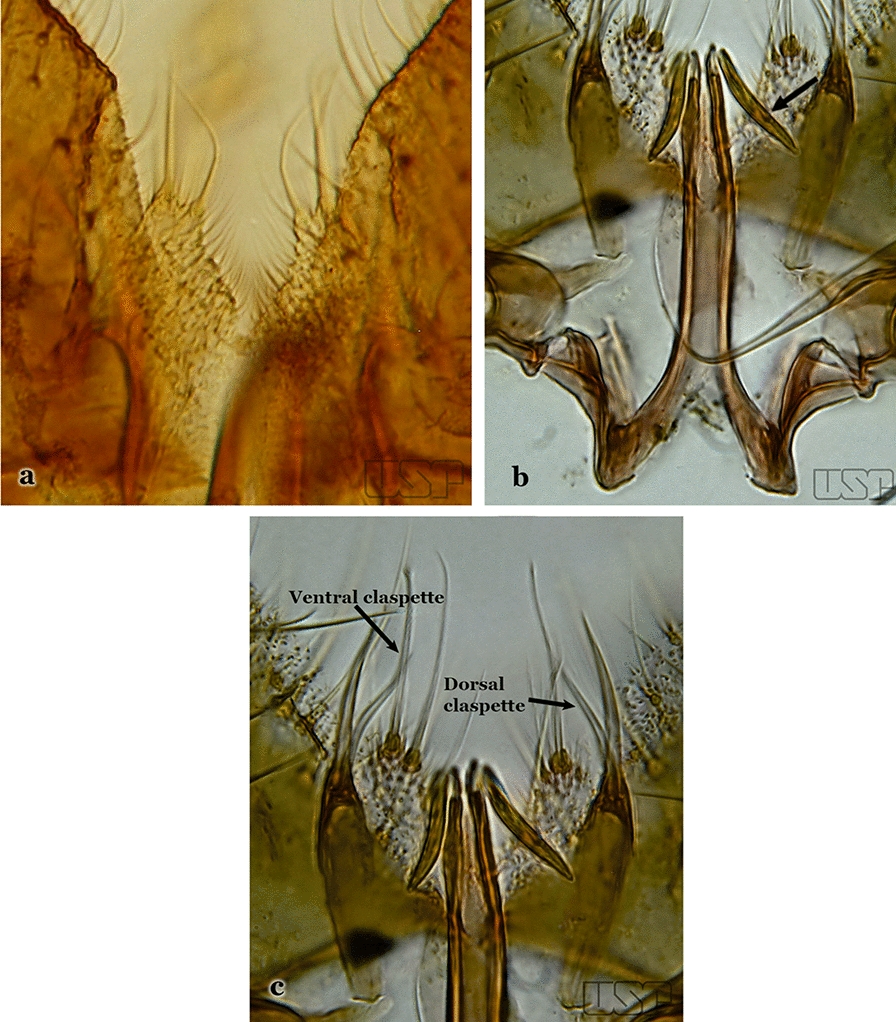
**a***An*. *mattogrossensis*. **b, c***An*. *eiseni geometricus* Correa, 1944
- Aedeagus with leaflets with serrate margins (Fig. 65b); dorsal claspette with 2 or 3 setae, one of which is longer and broader than others (Fig. 65c)…..*An. eiseni eiseni* & *An. eiseni geometricus*
- Gonocoxite without an internal seta (Fig. 66a)…..61

**Fig. 66:**
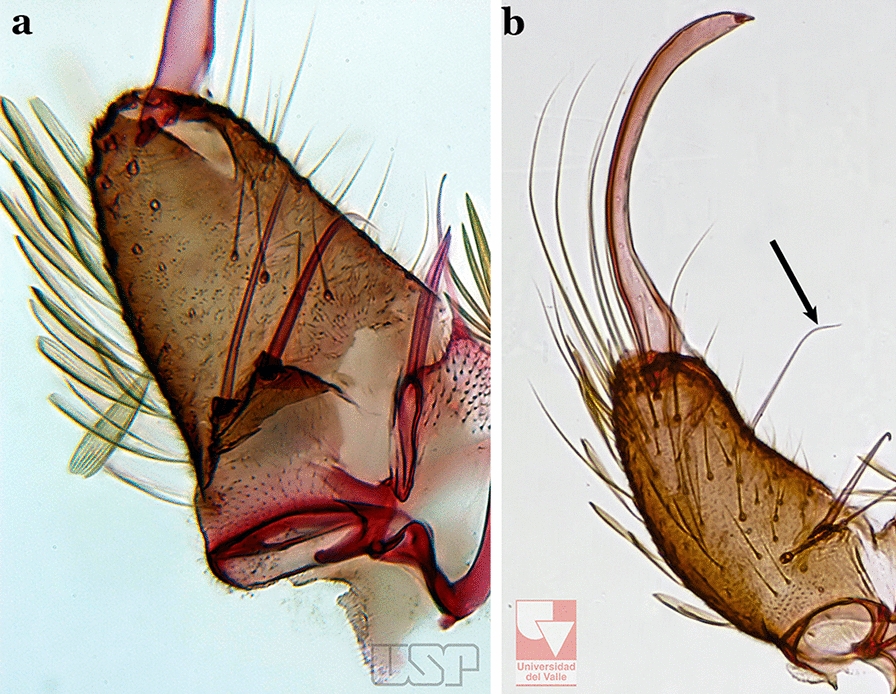
**a***An*. *peryassui*. **b***An*. *calderoni*
- Gonocoxite with an internal seta (Fig. 66b)…..62
- Aedeagus elongate, with 4 or more pairs of apical leaflets gradually decreasing in size, terminal leaflet well sclerotized (Fig. 67a); ventral claspette with a long apical seta (Fig. 67b)….. *An. peryassui*

**Fig. 67:**
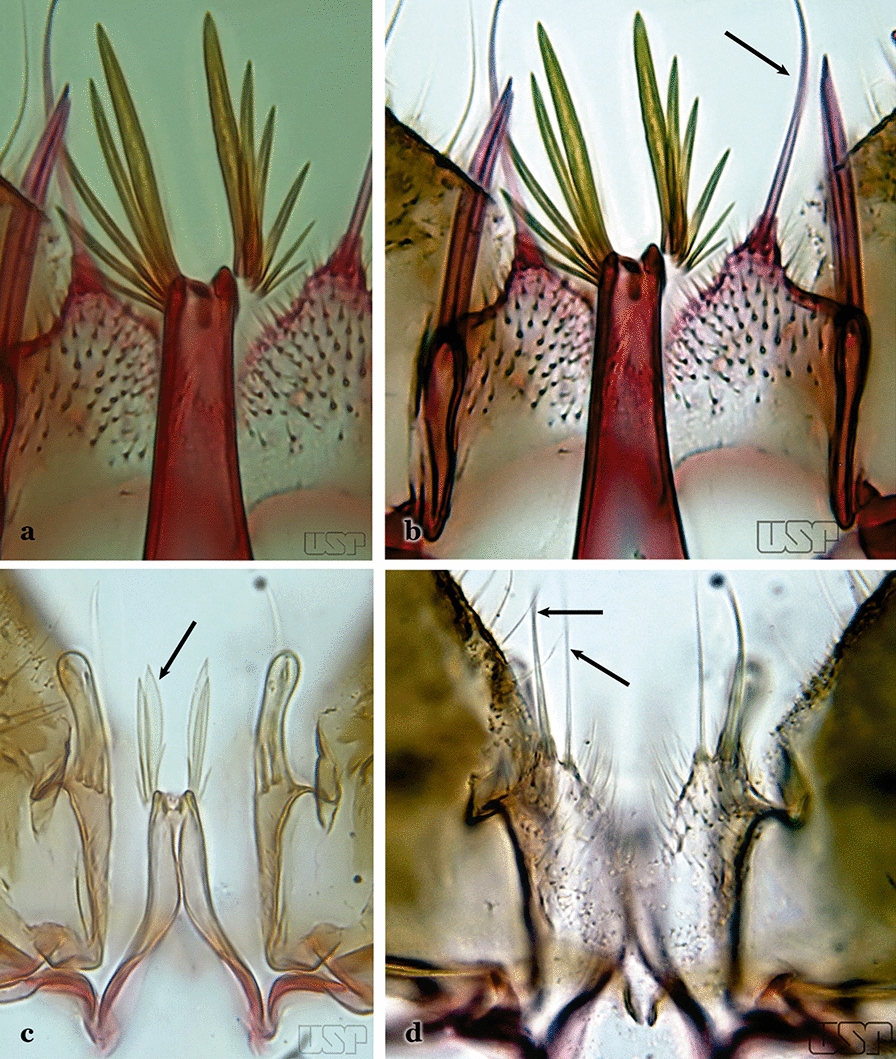
**a**, **b***An*. *peryassui*. **c, d***An*. *minor* da Costa Lima, 1929
- Aedeagus short, with 2 primary pairs of apical leaflets on each side and 2 or 3 rudimentary ones; ventral claspette with 2 long setae at apex (Figs. 67c, d)….. *An. minor*
- Aedeagus with a pair of broad apical leaflets, remaining leaflets indistinct, variable in number, resembling spicules (Fig. 68a)…..63

**Fig. 68:**
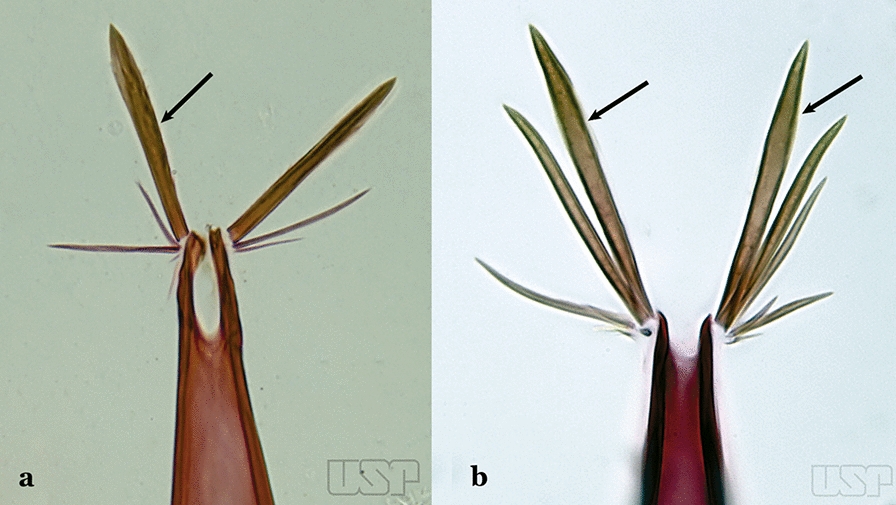
**a***An*. *anchietai* Corrêa & Ramalho, 1968. **b** *An*. *fluminensis* Root, 1927
- Aedeagus with a pair of apical leaflets, narrower than those described above or spiculate, remaining leaflets can vary in number and development, but are always easily visible (Fig. 68b)…..64
- Aedeagus with a pair of apical leaflets, size varying from 0.5 to 0.75 length of aedeagus; apical aperture wide and slightly concave (Fig. 69a)…..*An. maculipes*

**Fig. 69:**
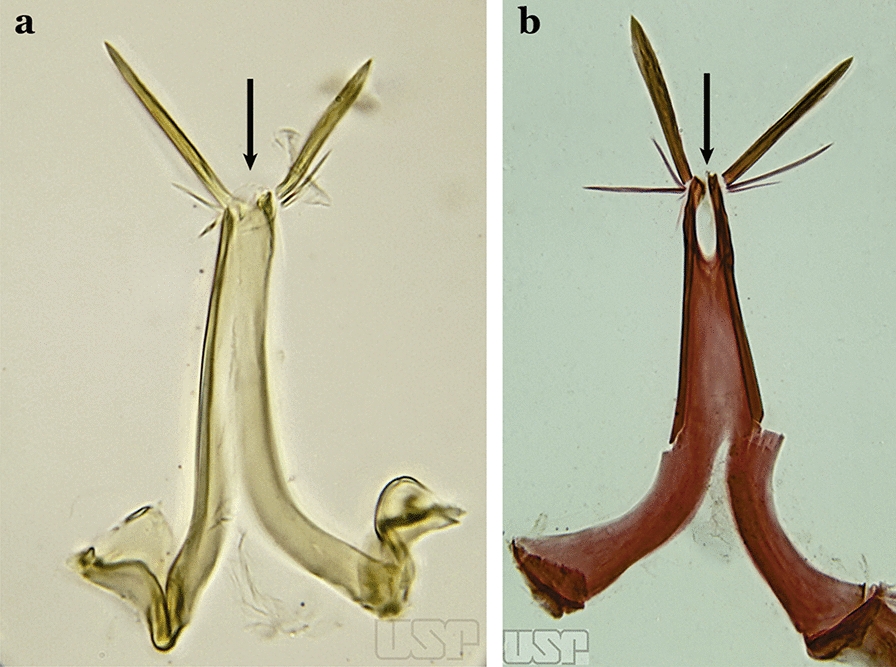
**a***An*. *maculipes* (Theobald, 1903). **b** *An*. *anchietai*
- Aedeagus with a pair of long and broad apical leaflets, length < 0.5 length of aedeagus; apical aperture narrow, elongated, deeply U-shape (Fig. 69b)….. *An. anchietai*
- Aedeagus with apical leaflets with smooth margins (Fig. 70a)…..65

**Fig. 70:**
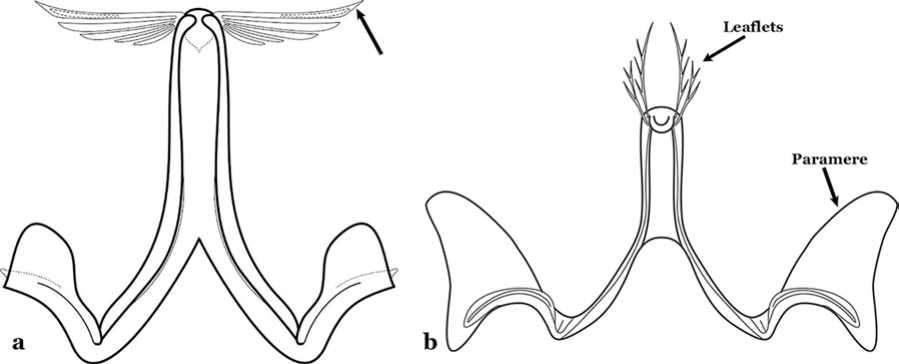
**a***An*. *apicimacula* Dyar & Knab, 1906. **b** *An*. *pseudopunctipennis* (both redrawn after Komp [7])
- Aedeagus with apical leaflets with margins distinctly serrated along their whole length (Fig. 70b)…..69
- Aedeagus relatively short, with a pair of long apical leaflets, straight with an oblique apex (Fig. 70a); ventral claspette with long spicules and 3 or 4 setae, one of them longer (Fig. 71a)….. *An. apicimacula*

**Fig. 71:**
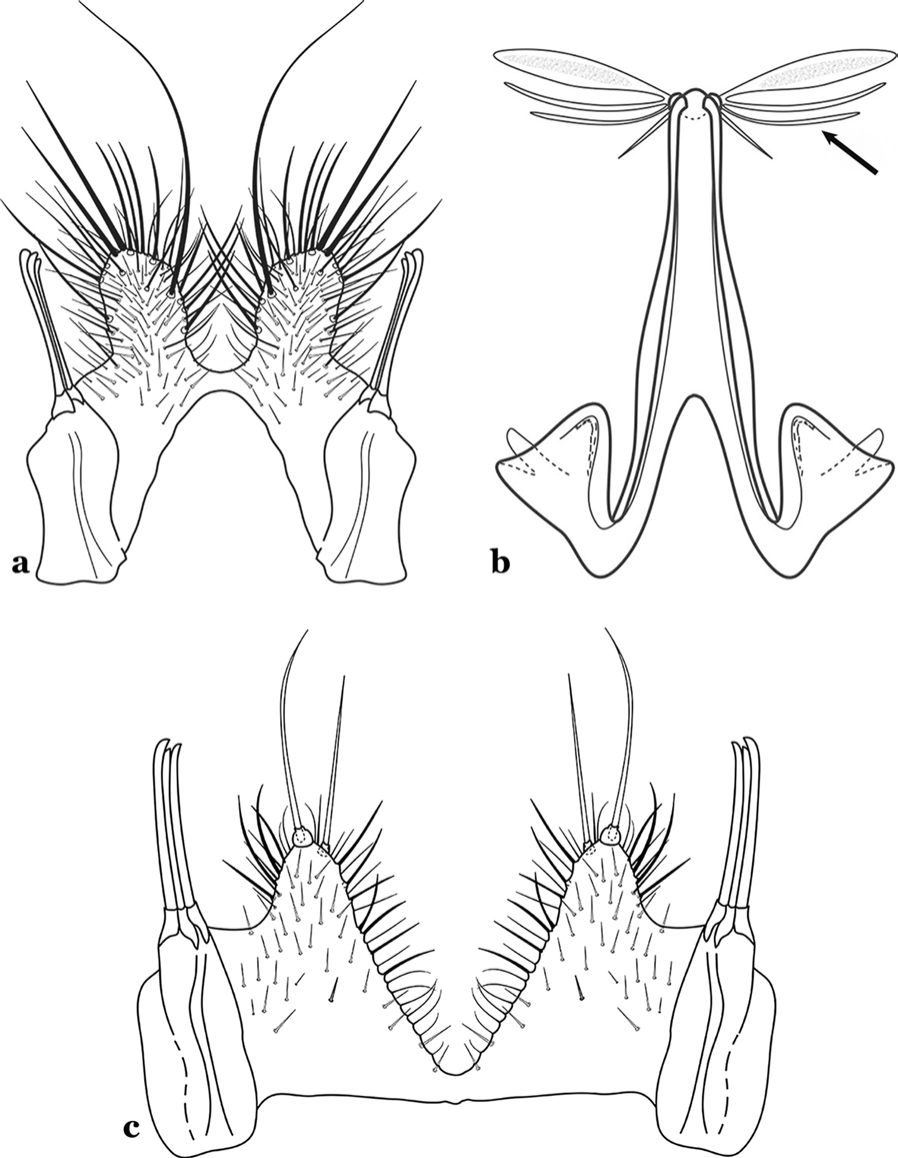
**a***An*. *apicimacula*. **b, c***An*. *punctimacula* Dyar & Knab, 1906 (both redrawn after Komp [7])
- Aedeagus of variable length, with 1 pair of apical leaflets more developed and broader (Fig. 71b); ventral lobe spicules of variable length and number (Fig. 71c)…..66
- Aedeagus with apical leaflets more sclerotized medially, margins hyaline (Fig. 72a); ventral claspette with a long slender and curved seta, and another smaller, slender one; dorsal claspette with a long stalk, with 3 strong well- developed setae inserted on apex, setae with curved and pointed apices (Fig. 72b)….. *An. calderoni*, *An. guarao*, *An. malefactor* & *An. punctimacula*

**Fig. 72:**
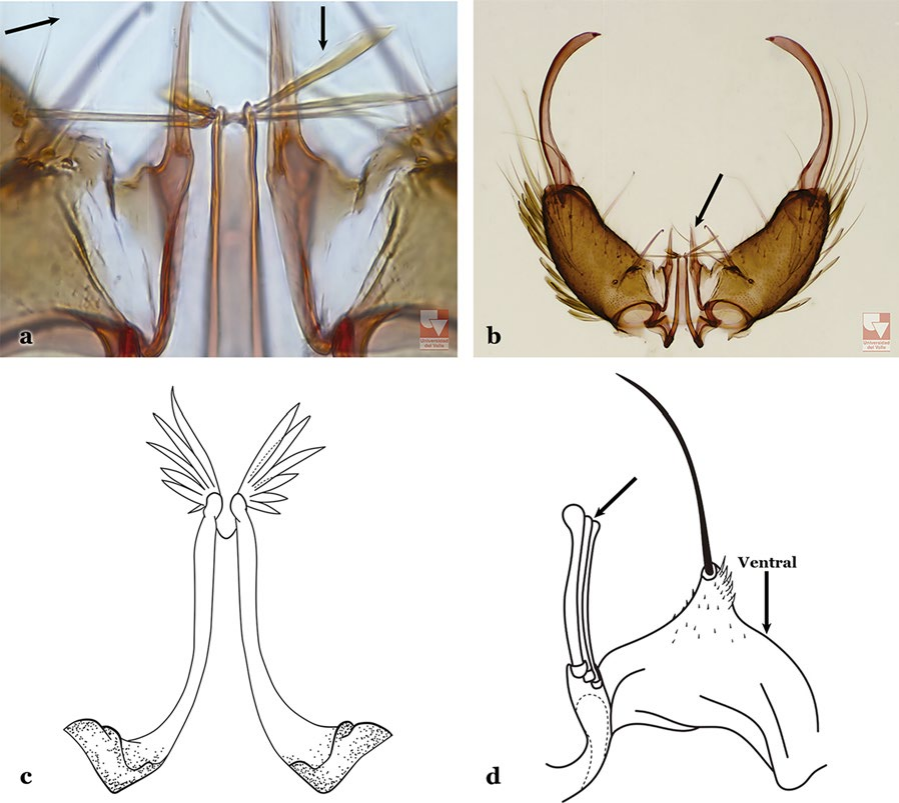
**a**, **b***An*. *calderoni* Wilkerson, 1991. **c**, **d** *An*. *shannoni* Davis, 1931 (redrawn after Wilkerson et al. [18])
- Aedeagus with a pair of uniformly sclerotized apical leaflets, including margins (Fig. 72c); ventral and dorsal claspette with variable (in number and form) setae inserted on apex (Fig. 72d)…..67
- Ventral claspette with a long apical seta (Fig. 72d); aedeagus with 5 to 8 pairs of apical leaflets (Fig. 72c)…..*An. shannoni*
- Ventral claspette with more than 1 pair of setae near apex (Fig. 73a); aedeagus with smaller number of apical leaflets (Fig. 73b)…..68

**Fig. 73:**
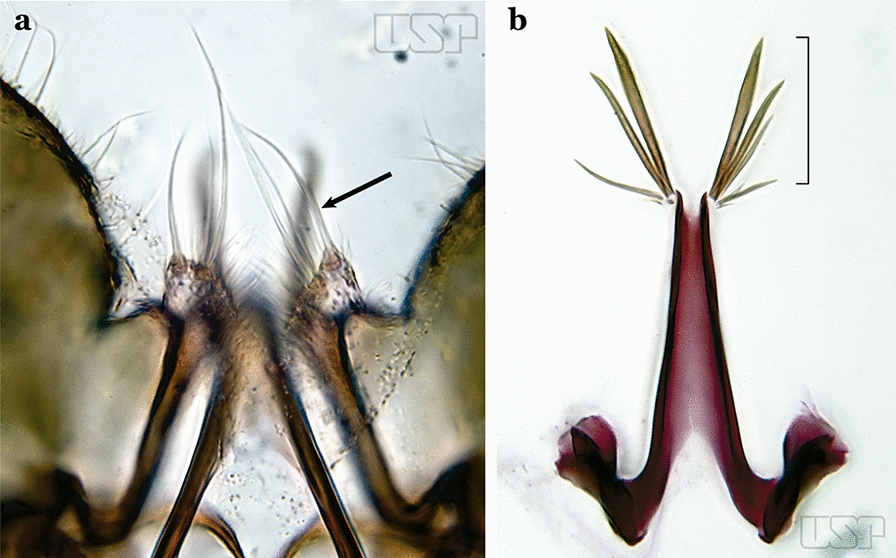
**a***An*. *medialis* Harbach, 2018 (redrawn after Wilkerson et al. [18]). **b** *An*. *fluminensis*
- Aedeagus with at least 3 pairs of small, uniformly sclerotized apical leaflets (Fig. 74a)…..*An. medialis*

**Fig. 74:**
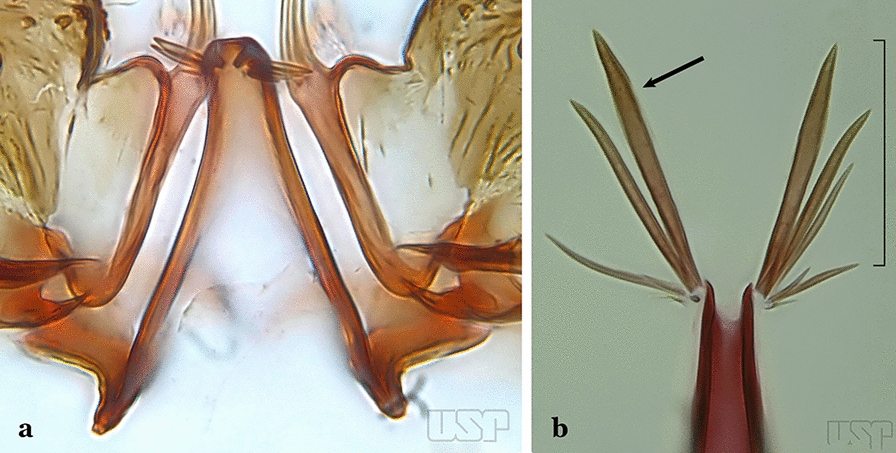
**a***An*. *medialis*. **b***An*. *fluminensis*
- Aedeagus with 5 pairs of long to moderately long apical leaflets with hyaline margins (Fig. 74b)…..*An. fluminensis*
- Aedeagus usually with 4 pairs of leaflets present, apical pair long, sabre- like, serrate or with small spicules at margins, widened beyond middle, tapering to apex, other leaflets slender (Fig. 75a); ventral claspette with 2 long, narrow setae; dorsal claspette with a short stalk, with 3 laminate setae, with apices expanded and slightly curved (Fig. 75b)…..*An. neomaculipalpus*

**Fig. 75:**
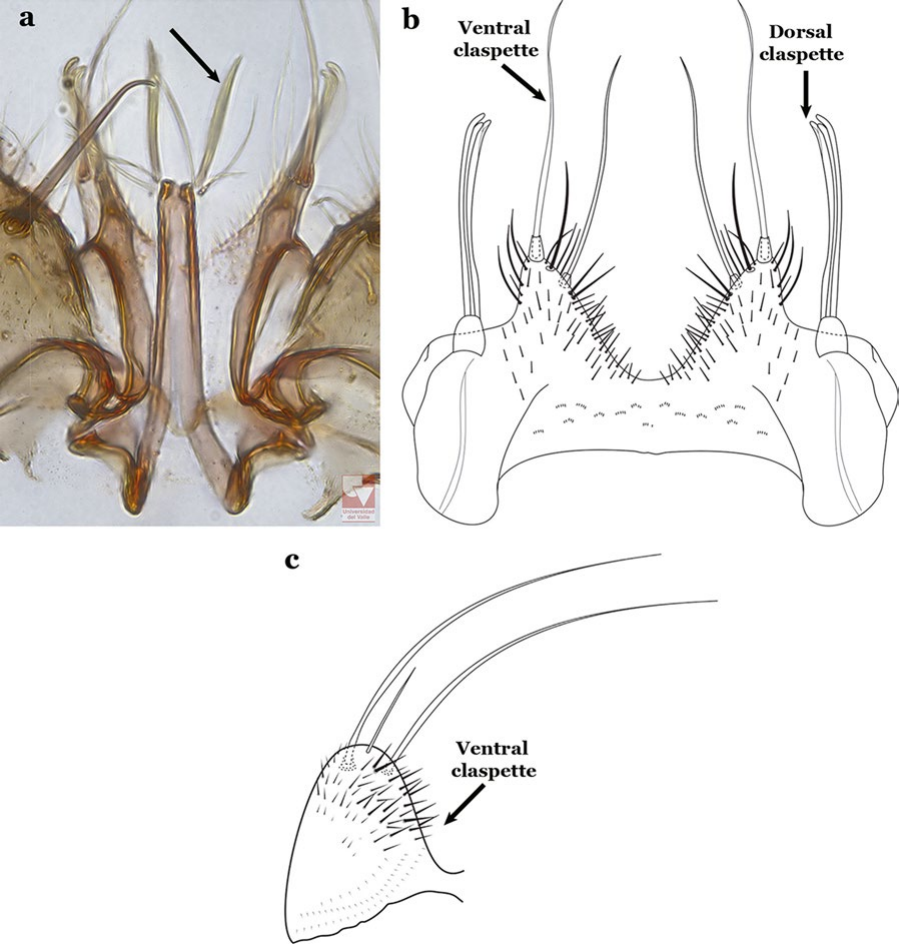
**a**, **b***An*. *neomaculipalpus* (**b** redrawn from [7], **c** *An*. *pseudopunctipennis* (redrawn after Komp [7])
- Aedeagus relatively short, strongly curved dorsally, with more than 1 pair of small, delicate apical leaflets (Fig. 70b); ventral claspette with 2 strong, long, curved setae (Fig. 75c); dorsal claspette with a columnar stalk, with 3 flattened, closely appressed, bladelike setae…..*An. pseudopunctipennis*

## Conclusions

Male genitalia possess characters that can be employed for accurate species identification. However, caution is necessary during the entire multi-step preparation procedure, including staining in a solution of acid fuchsine, separation of some parts with extra-fine needles, and repositioning each part on the microscope slide before covering with a coverslip. Any distortion during the dissection and mounting process presents an obstruction for accurate identification. In particular, the ventral claspette must not be distorted, because this will obstruct recognition of its unique features and shape that are essential for identification. For some species, the shape, and anatomical details of the aedeagus must be examined for species identification. For members of the Myzorhynchella Series, the ventral and dorsal claspettes possess multiple characteristics that are here employed for species identification.

## Data Availability

Specimens used in the current study are deposited and available in the Coleção Entomológica de Referência, Faculdade de Saúde Pública, Universidade de São Paulo (FSP-USP), São Paulo State, Brazil, the US National Mosquito Collection, Smithsonian Institution, Washington, DC, USA (USNMC), and the Facultad de Ciencias Naturales y Exactas de la Universidad del Valle, Colombia.

## References

[1] Sallum MAM, Gonzalez Obando R, Carrejo N, Wilkerson RC (2020). Identification keys to the *Anopheles* mosquitoes of South America (Diptera: Culicidae). I. Introduction. Parasit Vectors..

[2] Sallum MAM, Gonzalez Obando R, Carrejo N, Wilkerson RC (2020). Identification keys to the *Anopheles* mosquitoes of South America (Diptera: Culicidae). II. Fourth instar larvae.. Parasit Vectors..

[3] Sallum MAM, Gonzalez Obando R, Carrejo N, Wilkerson RC (2020). Identification keys to the *Anopheles* mosquitoes of South America (Diptera: Culicidae). IV. Adult females. Parasit Vectors..

[4] Harbach RE, Knight KL (1980). Taxonomistsʼ glossary of mosquito anatomy.

[5] Harbach RE, Knight KL (1981). Corrections and additions to taxonomistsʼ glossary of mosquito anatomy. Mosq Syst..

[6] McAlpine JF, Peterson BV, Shewell GE, Teskey HJ, Vockeroth JR, Wood DM (1981). Manual of Nearctic Diptera.

[7] Komp WHW (1942). The anopheline mosquitoes of the Caribbean Region. Natl Inst Health Bull.

[8] Forattini OP (1996). Culicidologia Médica. Vol. I. Principios gerais, morfologia, glossario taxonômico.

[9] Faran ME (1980). Mosquito studies (Diptera: Culicidae) XXXIV. A revision of the Albimanus section of the subgenus *Nyssorhynchus* of *Anopheles*. Contr Amer Entomol Inst..

[10] Faran ME, Linthicum KJ (1981). A handbook of the Amazonian species of *Anopheles* (*Nyssorhynchus*) (Diptera: Culicidae). Mosq Syst..

[11] Forattini OP (2002). Culicidologia Medica. Vol. II. Identificacão, Biologia, Epidemiologia.

[12] Komp WHW (1937). The species of the subgenus *Kerteszia* of *Anopheles* (Diptera, Culicidae). Ann Entomol Soc Am..

[13] Zavortink TJ (1973). Mosquito studies (Diptera, Culicidae) XXIX. A review of the subgenus *Kerteszia* of *Anopheles*. Contrib Amer Entomol Inst..

[14] Linthicum KJ (1988). A revision of the Argyritarsis Section of the subgenus *Nyssorhynchus* of *Anopheles* (Diptera: Culicidae). Mosq Syst..

[15] Levi-Castillo R (1955). Un nuevo anofelino de altura del Ecuador: *Anopheles gomezdelatorrei* n. sp. Revta Ecuator Ent Parasit..

[16] Lane J (1953). Neotropical Culicidae.

[17] Wilkerson RC, Sallum MAM (1999). *Anopheles* (*Anopheles*) *forattinii*: a new species in the Series Arribalzagia (Diptera: Culicidae). J Med Entomol..

[18] Wilkerson RC, Sallum MAM, Forattini OP (1997). Redescription of *Anopheles* (*Anopheles*) *shannoni* Davis; a member of the Arribalzagia Series from the Amazon Basin (Diptera: Culicidae). Proc Entomol Soc Wash.

